# Megabarcoding dark taxa – Assessing the utility of mass DNA barcoding for phorid fly species discovery

**DOI:** 10.1371/journal.pone.0334948

**Published:** 2025-12-02

**Authors:** Jiri Vihavainen, Niina Kiljunen, Pekka Pohjola, Jaclyn McKeown, Eveliina Oinonen, Marko Mutanen, Jaakko Pohjoismäki

**Affiliations:** 1 Department of Environmental and Biological Sciences, University of Eastern Finland, Joensuu, Finland; 2 Ecology and Genetics Research Unit, University of Oulu, Oulu, Finland; 3 Centre for Biodiversity Genomics, University of Guelph, Guelph, Ontario, Canada; Paris Lodron Universitat Salzburg Universitatsbibliothek Salzburg, AUSTRIA

## Abstract

Many hyperdiverse, small-bodied insect families contain numerous undescribed species, generally termed “dark taxa”. Scuttle flies (Diptera: Phoridae), being among the most diverse insect groups globally, are a prime example. DNA barcoding can help delineate dark taxa, particularly when integrated with morphology, and/or additional molecular evidence. We sequenced *COI*-barcodes from 9,120 Finnish phorid specimens and initially identified them using the BOLD database. Furthermore, species identifications of all 843 specimens, belonging to other genera than *Megaselia,* were confirmed morphologically. Initially, the BOLD-based identifications matched the morphological identifications only in 68% of the cases, primarily due to numerous misidentifications in BOLD. After adjusting the BOLD reference identifications based on morphological analyses of male features, we established a reliable framework for female identification. This is advantageous for future identification of females, as they are often excluded from traditional identification keys. Only two species were discovered as new to Finland, demonstrating that Finnish fauna is well-known, except *Megaselia*. Although DNA barcodes show great promise for identifying phorids, incorrectly identified reference sequences remain challenging, not the functionality of barcode gene *Cytochrome c oxidase subunit I* (COI) itself. The number of *Megaselia* BINs greatly exceeded the known Finnish species count, with many sequences lacking matches in BOLD. This further highlights *Megaselia* as a particularly dark group, for which genetic tools are essential for uncovering species identities and assessing diversity.

## Introduction

Despite over 260 years of effort in taxonomy, our grasp of the planet’s biodiversity is still just scratching the surface [1]. Alongside the ongoing anthropogenic biodiversity crisis, taxonomists are facing a taxonomic impediment, where inefficiency and lack of resources have slowed progress in species delimitation and taxonomic descriptions [2]. Meanwhile, the species extinction rate continues to exceed the emergence of new species, and the number of taxonomist experts continues to dwindle [2,3]. It is estimated that the world would need approximately 15,000 taxonomists to identify all life on Earth if based on traditional, primarily morphological practices. The current number of taxonomists is insufficient to document biodiversity, and their expertise is disproportionately focused on better-known groups of organisms. A notable example of this limitation is the nationwide inventory of insects in Sweden, which, despite an enormous two-decade effort, identified only 1% of the samples to the species level [4,5]. Molecular methods, such as DNA barcoding, could offer an efficient solution to overcoming the taxonomic impediment [3]. These methods have been successfully implemented for several groups previously considered challenging or even inaccessible with traditional morphological means [6–13]. While national and regional DNA barcoding initiatives can produce quantities of information, an ever-growing number of sequences cannot be assigned to the species level, especially those belonging to hyperdiverse, species-rich groups of small-bodied insects [14–17]. Groups with most species remaining undescribed have been termed “dark taxa”, often representing taxonomically speciose groups, such as Diptera and Hymenoptera [6]. While dark taxa may constitute the majority of all biodiversity in the tropics, they can also represent a significant portion of certain hymenopteran and dipteran families in Europe, despite the long history of taxonomic research in the subcontinent. One such recognized dark taxon are the scuttle flies (Diptera: Phoridae).

The scuttle flies have been proposed to be among the most diverse groups of insects with 4,400 described species worldwide [18], while 200,000 estimated species are just in the Afrotropical region alone [19]. Also, the larval feeding habits are exceptionally diverse [20,21]. While many scuttle flies are essential scavengers and decomposers of decaying organic matter, there are also many herbivorous, predatory and parasitoid species. For example, the genus *Pseudacteon* Coquillett, 1907 is infamous for parasitizing fire ants by decapitating them during their larval stage and using their empty head capsule for pupation [22]. Because of this feature, they have been used as a biological control agent in North America [23], although the introduction has not had significant successes in controlling the fire ant populations [24,25]. The most diverse genus of scuttle flies is *Megaselia* Rondani, 1856, with over 1,700 described species [18], with the majority of species remaining undescribed [13]. The *Megaselia* are considered an extraordinarily diverse genus, even among other species-rich insect taxa [6].

Identifying specimens into species using DNA barcodes has been highly successful across a variety of invertebrate groups, such as true bugs [26], chironomid midges [27], neuropterids [28], bees [29], beetles [30], tachinid flies [31], moths [32] and butterflies [33]. Intermediate results have been obtained for families considered to be taxonomically inaccessible, such as highly diverse flies and midges [34]. Several national barcode initiatives, for instance in Canada [35], Germany: German Barcode of Life Initiative [36], and in Finland: Finnish Barcode of Life [37] aim to generate DNA barcode reference libraries in respective countries, and some regional consortia (e.g., iBOL Europe, https://iboleurope.org) under the International Barcode of Life (https://ibol.org) research alliance work for the same goal over wider geographic regions.

Although DNA barcoding shows enormous promise for completing the inventory of life [3]. It has some shortcomings too [38–40]. Also, because DNA barcoding relies on reference sequences for identification, a sound and comprehensive reference library is required [3,40]. DNA barcoding was initially met with scepticism by traditional taxonomic societies, who feared it might undermine the intellectual depth of morphology-based taxonomy [41]. However, it was also quickly recognized that DNA barcoding is not a replacement for morphology-based taxonomy, but a complementary tool that also supports and enhances morphological identification [42].

Optimally, comprehensive DNA barcode libraries (e.g., [37,43]) should enable the identification of unknown specimens at the species level. This is not only required to facilitate the identification of individual specimens, but also for the purpose of species inventories conducted through metabarcoding [44]. In the case of specimens without a match in the database, operational taxonomic units (OTUs) such as those based on the Barcode Index Numbers system (BINs) can be used as units of putative species [45]. Besides the aforementioned issue with taxonomic impediment preventing the verification of the identity by morphological means, the utility of DNA barcoding for species identification is highly dependent on the reliability of the available databases. The problem is amplified if reference databases contain frequent misidentifications that result in subsequent specimens becoming misidentified and established in other databases through a circular identification process.

In the present work, we sought to elucidate the usability of DNA barcoding to identify Finnish scuttle flies (Diptera: Phoridae). To achieve this, we sequenced the full-length *COI* DNA barcodes for 9,120 scuttle fly specimens trapped throughout Finland and utilized the BOLD identification engine to obtain tentative identities for species already present in the database. Our focus was on scuttle flies other than *Megaselia*, as they are possible to morphologically identify to species level without years of thorough identification training and, therefore, can be used to confirm tentative species identities. Besides testing the *COI* ability to distinguish scuttle fly species, our combined approach also allowed us to curate and correct species identifications for these other genera in the BOLD database.

## Materials and methods

**Fig 1:**
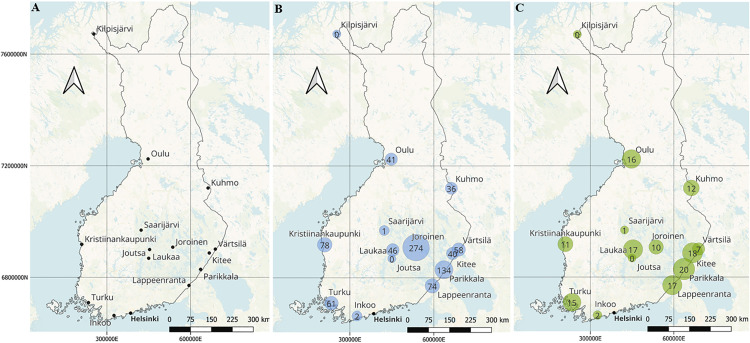
(A) Sampling sites across Finland. Sampling was done using 27 traps in 14 localities. Capitol city of Helsinki as a point of reference. **(B)** Captured and processed specimens in each locality represented by blue circles according to their abundance. **(C)** The count of captured and processed BIN diversity of each locality is represented by green circles depending on the abundance. Licence notice: Map tiles by CartoDB, under CC-BY 3.0. Data by OpenStreetMap, under ODbL. Coordinates are in ETRS-TM35FIN coordinate system. The maps were created in QGIS 3.34 Prizren [55].

Insect specimens were collected using malaise or window trap approaches with 80% to absolute ethanol or 70% glycol as collection liquid, respectively. The traps collect mainly flying insects and, therefore, are unlikely to catch wingless females of some phorid genera in high abundance. The sampling was done across Finland in 14 localities (Fig 1A) from May to October of 2015–2017 and 2020–2022. More detailed information about sampling is listed in the S1 Table. The scuttle flies (Diptera: Phoridae) were sorted from the insect material. Due to extremely high abundance of scuttle flies in this material, a randomized subset of 9,120 was selected. This was achieved by placing all sorted scuttle flies from each site and collecting period into separate petri dishes, from which individual specimens were randomly selected and placed in 96 microplates containing 30 microliters of 96% ethanol. The microplates were sent to the Canadian Centre for DNA Barcoding (CCDB), Guelph, Canada, where the samples were photographed and DNA barcoded for the *COI* gene marker (658 bp) using the LepF1 and LepR1 primers [46]. The barcoding was done using the CCDB pipeline for Sequel II sequencing [47].

The DNA-based identification of specimens was first done using the BOLD Identification Engine [48] with 98% K2P sequence similarity used as a proxy for preliminary species-level assignment. Each BIN identified only to the family level was further identified to the genus level using the BOLD Identification Engine with 90% K2P sequence similarity, and the genus assignments were verified using morphology. Subsequently, morphological identification of all species, except those of *Megaselia*, was performed using relevant identification keys [49–54], where applicable. Due to the lack of identification keys for females, particularly within the genus *Phora* Latreille, 1796, morphological analyses of females in this genus were limited to determining their sex. Additionally, 29 specimens across several genera were too damaged in order to reliably identify their sex. Female specimens of other genera were identified morphologically, alongside their male counterparts. For *Phora*, females were identified by matching their barcodes to those of morphologically verified males. Barcode-based inference was also applied to females of other genera and degraded specimens to validate the method and ensure accurate species identification. A total of 843 specimens were identified using this combined approach of morphological examination and barcode inference. Among these, one specimen produced a DNA barcode sequence matching *Metopina oligoneura* (Mik, 1867) but was unequivocally identified as *Phalacrotophora berolinensis* Schmitz, 1920 based on morphology, as the two genera are distinctly different. Consequently, this specimen was excluded from further analyses. The source of the contamination remains unclear, as the morphological examination was performed on the sequenced specimen.

## Results

**Fig 2:**
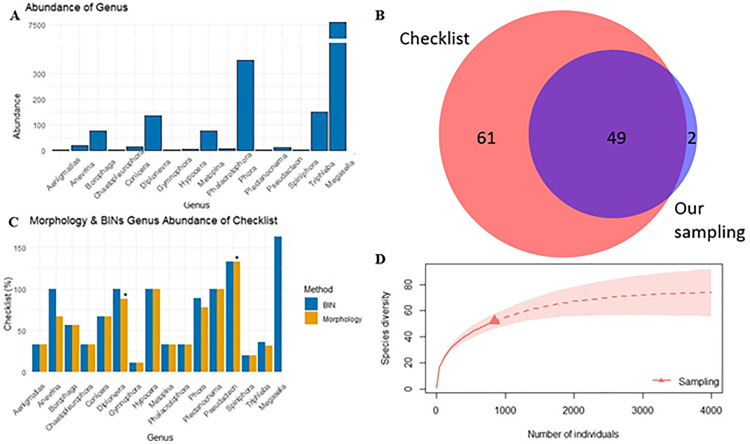
(A) Genus-level representation of the specimens from our sampling. Note how *Megaselia* specimens significantly outnumber other genera. (B) Venn diagram with known Finnish phorid fauna, except *Megaselia*, and species found in our sampling. The overlap area represents 46% of the known Finnish species. (C) A diagram showing how well each genus was described in the malaise trap samples, compared to the species list of Phoridae in the FinBIF database (%). Species identified using morphology or BINs are shown separately. Note that *Megaselia* specimens were not morphologically examined. Some morphologically identified species might have more than one BIN. * Genera with new species to Finland. (D) Rarefaction plot to estimate the species richness of phorid species other than *Megaselia* of Finland.

**Fig 3:**
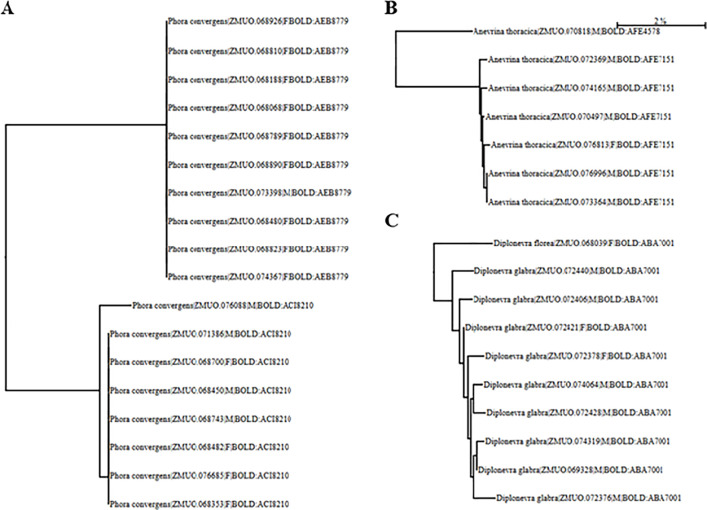
Showcase of NJ tree with examples of species sharing multiple BINs (A & B), and two species sharing the identical BIN (C). **(A)** *Phora convergens* Schmitz, 1920 is split into two BINs, but no geographical pattern was detected. **(B)** *Anevrina thoracica* is split into two BINs. **(C)** *Diplonevra florea* and *D. glabra* share the identical BIN, although morphological analyses revealed them to represent two separate morphological species. Scale bar: 2% difference.

Out of 9,120 specimens analysed, 8,363 yielded a high-quality DNA barcode (>600 bp without stop codons) suitable for further analysis. Validated sequences were grouped into 424 BINS, with the most common genus being *Megaselia* (Fig 2A). BOLD Identification Engine with 98% similarity, assigned 6,014 specimens (72%) to species level, representing to 171 species (S2 Table). Of these, 43 were species belonging to other genera than *Megaselia*, comprising 798 specimens. The remaining family-level BINs were further identified to a genus level with 90% similarity, bringing the total number of specimens to 843 across 55 BINs. Morphological identification confirmed 15 genera and 51 species, excluding one contaminant. These included 18 species represented by only one single specimen (singletons), while the most common species, *Phora pubipes* Schmitz, 1920, accounted for 15% of the samples. In some instances, two BINs were found within a single morphological species (Fig 3A & B). This oocurred with *Anevrina thoracica* (Meigen, 1804), *Phora obscura* (Zetterstedt, 1848), *P. artifrons* Latreille, 1796, and *Triphleba nudipalpis* (Becker, 1901). Meanwhile, a shared BIN was observed between *Diplonevra florea* (Fabricius, 1794) and *D. glabra* Schmitz, 1927 (Fig 3C). Initial barcode-based identification achieved a 68% success rate for correct species assignment, prior to the incorporation of morphological corrections into the BOLD database. The morphological analyses enhanced the accuracy of BOLD reference database, increasing the identification success to 100%.

Cross-referencing the determined species against the known Finnish fauna [56,57] revealed our sampling to cover 70% of known Finnish genera (Table 1) and 46% of the species which belong to other genera than *Megaselia* (Fig 2B, made with DeepVenn [58]). The morphological analyses verified two new species for Finland: *Diplonevra florea*, and *Pseudacteon brevicauda* Schmitz, 1925. Each genus was compared against the known checklist and database of Finnish species as a bar plot (Fig 2C). In the comparison, the BIN count is slightly higher due to some species containing multiple BINs.

**Table 1:** Genus representation among the samples.

The Finnish checklist	Malaise trap samples
*Abaristophora*	Not found
*Aenigmatias*	Present
*Anevrina*	Present
*Borophaga*	Present
*Chaetopleurophora*	Present
*Conicera*	Present
*Diplonevra*	Present
*Dohrniphora*	Not found
*Gymnophora*	Present
*Gymnoptera*	Not found
*Hypocera*	Present
*Megaselia*	Present
*Menozziola*	Not found
*Metopina*	Present
*Microselia*	Not found
*Phalacrotophora*	Present
*Phora*	Present
*Plectanocnema*	Present
*Pseudacteon*	Present
*Spiniphora*	Present
*Triphleba*	Present
*Trucidophora*	Not found
*Veruanus*	Not found

The DNA barcodes effectively distinguish the species of all other genera. The mean minimum of K2P divergence to the nearest neighbour is 9.35% (range 1.4–20.9%, SD = 3.46, SE = 0.07), which is higher than reported for Tachinidae (5.51% with 366 species, [31]), but lower than in Coleoptera (11.99% with 1872 species, [30]), but it should be noted that the latter includes multiple families. Similarly, the relatively significant K2P divergence in our sample can be attributed to differences between genera, as divergences within species-rich genera with recent radiations, such as Tachinidae [59], tend be smaller. For example, less than 2% divergence was observed between *Diplonevra glabra* and *D. florea*.

A rarefaction plot (Fig 2D) was generated to estimate the efficiency of our sampling effort. The curve shows that while our sampling effort rapidly covered half of the species, more remain to be discovered, as evidenced also by the number of singleton observations. Our sampling covered 51 species (Fig 2B), and the extrapolations from the rarefaction plot would estimate the Finnish species count for other genera than *Megaselia* to slightly exceed 100 species (Fig 2D). Considering that 110 species from these genera were previously known from Finland (Fig 2B), this further highlights the comprehensive current knowledge of the country’s fauna.

## Discussion

To elucidate the utility of DNA barcoding for species inventories of hyperdiverse taxa, we sequenced a geographically representative sample of 9,120 phorid fly specimens from Finland. The barcoding was successful for 8,363 specimens, with samples collected in ethanol yielding the best results. 7,520 of the specimens belong to the genus *Megaselia* and 843–15 other genera of Phoridae, representing 70% of all genera reported from Finland (Table 1) (S3 Table). The species identifications for the 843 specimens, representing 55 BINs, were verified by morphologically determining the males (all genera) and females (excluding genus *Phora*), and then inferring the species identities of *Phora* females based on matching male barcodes. This method was also applied to all females to validate its accuracy. Using this approach, a total of 51 scuttle fly species were identified. Since the barcodes were able to distinguish the males of different species unambiguously, we argue that the same approach can be reliably used to identify females. This is particularly important in the contexts such as biomonitoring, where females of many genera are usually overlooked due to identification difficulties. Additionally, this approach provides new opportunities to examine and identify morphological differences between females and aids in developing new morphological identification keys. Interestingly, *Phora convergens* is divided into two BINs, which may represent two distinct but morphologically cryptic species (Fig 3A). Apart from *P. convergens*, there is only one known species with a similar right epandrial appendage, namely *P. indivisa* Schmitz, 1948 known from the Alps and described based on a single specimen [54]. However, the specimens do not morphologically match its species description. Sequencing of nuclear markers would probably provide the most effective way to determine if the name *P. convergens* currently contains two biologically distinct species. Other species with multiple BINs include *Anevrina thoracica* (Fig 3B), *Phora obscura*, *P. artifrons*, and *Triphleba nudipalpis*. Conversely, a shared BIN was observed between *Diplonevra florea* and *D. glabra*, although our results show that the two can also be distinguished by the clustering of their barcodes on the NJ-tree (Fig 3C).

Despite including only 14 localities, our sampling reached 46% of the known Finnish fauna, when *Megaselia* is excluded. Only two species (*Diplonevra florea* and *Pseudacteon brevicauda*) were new to Finland. This makes Finnish fauna well known, continuing a trend seen in the rest of Europe where new species belonging to these other genera are found scarcely [6,60]. Also, in our sampling, the collection was mainly focused to the central and eastern parts of Finland, with only few sites on the coastline (Fig 1B & 1C). With our current sampling, the rarefaction curve did not reach an asymptote (Fig 2D), which is likely because our sampling failed to capture several known genera. Then again, the sampling discovered two new species in Finland, indicating the importance of adequate sampling effort needed with this taxon. It is evident from singleton observations that many species are either rare or inhabit a different habitat from those we sampled. Evidently, the genus *Megaselia* is where the majority of new species of Phoridae remain to be discovered. For example, the current FinBIF database contains 226 *Megaselia* species, whereas the BIN count of 369 suggests the true species count is at least a third higher or even more. Previous studies have revealed a similar trend. For example, in the neighbouring country of Sweden, 500 new scuttle fly species were found [13], while in Germany an increase of 12% of species was reported [61]. Given the high performance of DNA barcoding in differentiating species of other genera, they could establish a robust framework for species discovery and further taxonomic research, also for the *Megaselia*, although this requires verification. This genus is notorious for many taxonomic difficulties [6,14,25,61], rendering species identification and discovery extremely laborious. One solution could be to complement DNA barcodes with nuclear loci to further validate species identities.

The scuttle fly species count in Sweden has been estimated to be around 652–713 [13]. The actual count is difficult to estimate, as scuttle flies’ ecology is very diverse, and it seems that rarer species also inhabit specific environments or microhabitats, while the most common species can be found with ease. This is also supported by our sampling, which captured 18 singleton species, many of which inhabited different areas. The most abundant species in our material was *Phora pubipes* with 130 specimens, followed by *P. tincta* Schmitz, 1920 with 117 specimens, the two altogether covering 29% of all the morphologically identified specimens.

Several studies [25,62–64] have concluded that no single sampling method can capture the full range of taxa, as the discovery of many species depends on using specific trapping techniques. Therefore, a more comprehensive sampling would require enormous effort and several trap types to provide an accurate estimate of species count. In this regard, new species will most likely be discovered in future monitoring surveys and by lucky accidents.

Initially, the barcode success rate to identify specimens correctly was as low as 68%, which, however, turned out to be due to many misidentifications in the reference database rather than poor performance of DNA barcoding. After re-examining the problematic reference specimens using morphological analysis, the identification success rate increased to 100%, indicating that the primary source of error was misidentified reference specimens. The impact of such misidentifications and other operational factors on the success of DNA barcoding has been previously examined in detail [65]. As a conclusion, the DNA barcodes perform well in identifying species of Phoridae. To this end, it is evident that a comprehensive and validated reference database is a necessity to bring Phoridae and other massively diverse dark taxa into efficient biomonitoring.

## Supporting information

S1 Table Comprehensive specimen details. Specimen details containing sample ID, BIN, species, sampling site, coordinates, collection date and trap type used (WiT = Window Trap, MaT = Malaise Trap).
(PDF)

S2 Table Initial BOLD Identification Engine results. Species identified using BOLD Identification Engine with >98% match, with the number of specimens from which *COI* barcode was recovered. Retrieved November 2023.
(PDF)

S3 Table Known species representation among samples. (PDF)
