# Supplementary material for: Megabarcoding dark taxa – Assessing the utility of mass DNA barcoding for phorid fly species discovery
Source: PLoS One. 2025 Dec 2;20(12):e0334948. doi: 10.1371/journal.pone.0334948 (PMC12671794; doi:10.1371/journal.pone.0334948)
Supplement: S1 Table — Specimen details containing sample ID, BIN, species, sampling site, coordinates, collection date and trap type used (WiT = Window Trap, MaT = Malaise Trap). (PDF) [file pone.0334948.s001.pdf]

S-Table 1. Comprehensive specimen details. Specimen details containing sample ID, BIN, species, sampling site, coordinates, collection date and trap type used (WiT = Window Trap, MaT = Malaise Trap).

Sample ID	BIN	Species	Site	Lat	Lon	Date	Trap
ZMUO.068002	BOLD:ABA7002	*Phora tincta*	Joroinen	62.301	27.699	27-Jun-2021	WiT
ZMUO.068004	BOLD:AFI7403	*Phora artifrons*	Joroinen	62.301	27.699	27-Jun-2021	WiT
ZMUO.068005	BOLD:ABA7002	*Phora tincta*	Joroinen	62.301	27.699	27-Jun-2021	WiT
ZMUO.068007	BOLD:ACQ9655	*Anevrina unispinosa*	Joroinen	62.301	27.699	27-Jun-2021	WiT
ZMUO.068009	BOLD:ABA7002	*Phora tincta*	Joroinen	62.301	27.699	27-Jun-2021	WiT
ZMUO.068010	BOLD:ACD2261	*Phora artifrons*	Joroinen	62.301	27.699	27-Jun-2021	WiT
ZMUO.068013	BOLD:ABA7002	*Phora tincta*	Joroinen	62.301	27.699	27-Jun-2021	WiT
ZMUO.068014	BOLD:ABA7002	*Phora tincta*	Joroinen	62.301	27.699	27-Jun-2021	WiT
ZMUO.068016	BOLD:ABA7002	*Phora tincta*	Joroinen	62.301	27.699	27-Jun-2021	WiT
ZMUO.068019	BOLD:ABA7002	*Phora tincta*	Joroinen	62.301	27.699	27-Jun-2021	WiT
ZMUO.068026	BOLD:ABA7002	*Phora tincta*	Joroinen	62.301	27.699	27-Jun-2021	WiT
ZMUO.068028	BOLD:ACD2261	*Phora artifrons*	Joroinen	62.304	27.71	27-Jun-2021	WiT
ZMUO.068034	BOLD:ACD2261	*Phora artifrons*	Joroinen	62.304	27.71	27-Jun-2021	WiT
ZMUO.068035	BOLD:ABA7002	*Phora tincta*	Joroinen	62.304	27.71	27-Jun-2021	WiT
ZMUO.068037	BOLD:ABA7002	*Phora tincta*	Joroinen	62.304	27.71	27-Jun-2021	WiT
ZMUO.068038	BOLD:ABA7002	*Phora tincta*	Joroinen	62.304	27.71	27-Jun-2021	WiT
ZMUO.068039	BOLD:ABA7001	*Diplonevra abdominalis*	Joroinen	62.304	27.71	27-Jun-2021	WiT
ZMUO.068046	BOLD:ABA7002	*Phora tincta*	Joroinen	62.304	27.71	27-Jun-2021	WiT
ZMUO.068050	BOLD:ACD2261	*Phora artifrons*	Joroinen	62.304	27.71	27-Jun-2021	WiT
ZMUO.068054	BOLD:ABA7002	*Phora tincta*	Joroinen	62.304	27.71	27-Jun-2021	WiT
ZMUO.068055	BOLD:ACD2261	*Phora artifrons*	Joroinen	62.304	27.71	27-Jun-2021	WiT
ZMUO.068058	BOLD:ACD2261	*Phora artifrons*	Joroinen	62.304	27.71	27-Jun-2021	WiT
ZMUO.068062	BOLD:ABA7002	*Phora tincta*	Joroinen	62.306	27.706	27-Jun-2021	WiT
ZMUO.068064	BOLD:ACN6664	*Phora pubipes*	Joroinen	62.306	27.706	27-Jun-2021	WiT
ZMUO.068066	BOLD:ACN6664	*Phora pubipes*	Joroinen	62.306	27.706	27-Jun-2021	WiT
ZMUO.068068	BOLD:AEB8779	*Phora convergens*	Joroinen	62.306	27.706	27-Jun-2021	WiT
ZMUO.068069	BOLD:ABA7002	*Phora tincta*	Joroinen	62.306	27.706	27-Jun-2021	WiT
ZMUO.068075	BOLD:ACN6664	*Phora pubipes*	Joroinen	62.306	27.706	27-Jun-2021	WiT
ZMUO.068076	BOLD:ABA7002	*Phora tincta*	Joroinen	62.306	27.706	27-Jun-2021	WiT
ZMUO.068077	BOLD:ABA7002	*Phora tincta*	Joroinen	62.306	27.706	27-Jun-2021	WiT
ZMUO.068085	BOLD:ACN6664	*Phora pubipes*	Joroinen	62.306	27.706	27-Jun-2021	WiT
ZMUO.068092	BOLD:ABA7002	*Phora tincta*	Joroinen	62.306	27.706	27-Jun-2021	WiT
ZMUO.068100	BOLD:ABA7002	*Phora tincta*	Joroinen	62.306	27.706	27-Jun-2021	WiT
ZMUO.068102	BOLD:AEB8779	*Phora convergens*	Joroinen	62.306	27.706	27-Jun-2021	WiT
ZMUO.068104	BOLD:ACN6664	*Phora pubipes*	Joroinen	62.306	27.706	27-Jun-2021	WiT
ZMUO.068105	BOLD:ABA7002	*Phora tincta*	Joroinen	62.306	27.706	27-Jun-2021	WiT
ZMUO.068109	BOLD:ACN6664	*Phora pubipes*	Joroinen	62.306	27.706	27-Jun-2021	WiT
ZMUO.068111	BOLD:ACN6664	*Phora pubipes*	Joroinen	62.306	27.706	27-Jun-2021	WiT
ZMUO.068118	BOLD:ACN6664	*Phora pubipes*	Joroinen	62.306	27.706	27-Jun-2021	WiT
ZMUO.068124	BOLD:ABA7002	*Phora tincta*	Joroinen	62.306	27.706	27-Jun-2021	WiT
ZMUO.068126	BOLD:AEB8779	*Phora convergens*	Joroinen	62.306	27.706	27-Jun-2021	WiT
ZMUO.068128	BOLD:ABA7002	*Phora tincta*	Joroinen	62.306	27.706	27-Jun-2021	WiT

ZMUO.068129	BOLD:ABA7002	*Phora tincta*	Joroinen	62.306	27.706	27-Jun-2021	WiT
ZMUO.068130	BOLD:ABA7002	*Phora tincta*	Joroinen	62.306	27.706	27-Jun-2021	WiT
ZMUO.068141	BOLD:ABA7002	*Phora tincta*	Joroinen	62.306	27.706	27-Jun-2021	WiT
ZMUO.068153	BOLD:ACN6664	*Phora pubipes*	Joroinen	62.306	27.706	27-Jun-2021	WiT
ZMUO.068159	BOLD:ABA7002	*Phora tincta*	Joroinen	62.306	27.706	27-Jun-2021	WiT
ZMUO.068162	BOLD:ABA7002	*Phora tincta*	Joroinen	62.306	27.706	27-Jun-2021	WiT
ZMUO.068165	BOLD:ABA7002	*Phora tincta*	Joroinen	62.306	27.706	27-Jun-2021	WiT
ZMUO.068168	BOLD:ABA7002	*Phora tincta*	Joroinen	62.306	27.706	27-Jun-2021	WiT
ZMUO.068170	BOLD:ACN6664	*Phora pubipes*	Joroinen	62.306	27.706	27-Jun-2021	WiT
ZMUO.068174	BOLD:ACN6664	*Phora pubipes*	Joroinen	62.306	27.706	27-Jun-2021	WiT
ZMUO.068180	BOLD:ABA7002	*Phora tincta*	Joroinen	62.306	27.706	27-Jun-2021	WiT
ZMUO.068188	BOLD:AEB8779	*Phora convergens*	Joroinen	62.306	27.706	27-Jun-2021	WiT
ZMUO.068189	BOLD:ABA7002	*Phora tincta*	Joroinen	62.306	27.706	27-Jun-2021	WiT
ZMUO.068190	BOLD:ACN6664	*Phora pubipes*	Joroinen	62.306	27.706	27-Jun-2021	WiT
ZMUO.068192	BOLD:ACN6664	*Phora pubipes*	Joroinen	62.306	27.706	27-Jun-2021	WiT
ZMUO.068193	BOLD:ABA7002	*Phora tincta*	Joroinen	62.306	27.706	27-Jun-2021	WiT
ZMUO.068197	BOLD:ABA7002	*Phora tincta*	Joroinen	62.306	27.706	27-Jun-2021	WiT
ZMUO.068202	BOLD:ABA7002	*Phora tincta*	Joroinen	62.306	27.706	27-Jun-2021	WiT
ZMUO.068208	BOLD:ACN6664	*Phora pubipes*	Joroinen	62.306	27.706	27-Jun-2021	WiT
ZMUO.068213	BOLD:ACN6664	*Phora pubipes*	Joroinen	62.306	27.706	27-Jun-2021	WiT
ZMUO.068225	BOLD:ABA7002	*Phora tincta*	Joroinen	62.306	27.706	27-Jun-2021	WiT
ZMUO.068229	BOLD:ACN6664	*Phora pubipes*	Joroinen	62.306	27.706	27-Jun-2021	WiT
ZMUO.068234	BOLD:ACN6664	*Phora pubipes*	Joroinen	62.306	27.706	27-Jun-2021	WiT
ZMUO.068239	BOLD:AFE7212	*Phora holosericea*	Joroinen	62.306	27.706	27-Jun-2021	WiT
ZMUO.068241	BOLD:AEB8779	*Phora convergens*	Joroinen	62.306	27.706	27-Jun-2021	WiT
ZMUO.068242	BOLD:ABA7002	*Phora tincta*	Joroinen	62.306	27.706	27-Jun-2021	WiT
ZMUO.068245	BOLD:ACN6664	*Phora pubipes*	Joroinen	62.306	27.706	27-Jun-2021	WiT
ZMUO.068246	BOLD:ABA7002	*Phora tincta*	Joroinen	62.306	27.706	27-Jun-2021	WiT
ZMUO.068250	BOLD:ABA7002	*Phora tincta*	Joroinen	62.306	27.706	27-Jun-2021	WiT
ZMUO.068251	BOLD:ACN6664	*Phora pubipes*	Joroinen	62.306	27.706	27-Jun-2021	WiT
ZMUO.068259	BOLD:ACN6664	*Phora pubipes*	Joroinen	62.306	27.706	27-Jun-2021	WiT
ZMUO.068271	BOLD:ABA7002	*Phora tincta*	Joroinen	62.306	27.706	27-Jun-2021	WiT
ZMUO.068272	BOLD:ABA7002	*Phora tincta*	Joroinen	62.306	27.706	27-Jun-2021	WiT
ZMUO.068273	BOLD:ACQ9655	*Anevrina unispinosa*	Joroinen	62.306	27.706	27-Jun-2021	WiT
ZMUO.068276	BOLD:ACN6664	*Phora pubipes*	Joroinen	62.306	27.706	27-Jun-2021	WiT
ZMUO.068288	BOLD:ABA7002	*Phora tincta*	Joroinen	62.306	27.706	27-Jun-2021	WiT
ZMUO.068290	BOLD:ABA7002	*Phora tincta*	Joroinen	62.306	27.706	27-Jun-2021	WiT
ZMUO.068291	BOLD:AEB8779	*Phora convergens*	Joroinen	62.306	27.706	27-Jun-2021	WiT
ZMUO.068292	BOLD:ACN6664	*Phora pubipes*	Joroinen	62.306	27.706	27-Jun-2021	WiT
ZMUO.068294	BOLD:ABA7002	*Phora tincta*	Joroinen	62.306	27.706	27-Jun-2021	WiT
ZMUO.068297	BOLD:ABA7002	*Phora tincta*	Joroinen	62.306	27.706	27-Jun-2021	WiT
ZMUO.068303	BOLD:ABA7002	*Phora tincta*	Joroinen	62.306	27.706	27-Jun-2021	WiT
ZMUO.068309	BOLD:ABA7002	*Phora tincta*	Joroinen	62.306	27.706	27-Jun-2021	WiT
ZMUO.068311	BOLD:ABA7002	*Phora tincta*	Joroinen	62.306	27.706	27-Jun-2021	WiT
ZMUO.068313	BOLD:ACN6664	*Phora pubipes*	Joroinen	62.306	27.706	27-Jun-2021	WiT
ZMUO.068316	BOLD:ACN6664	*Phora pubipes*	Joroinen	62.306	27.706	27-Jun-2021	WiT
ZMUO.068318	BOLD:ABA7002	*Phora tincta*	Joroinen	62.306	27.706	27-Jun-2021	WiT
ZMUO.068321	BOLD:ACN6664	*Phora pubipes*	Joroinen	62.306	27.706	27-Jun-2021	WiT

ZMUO.068328	BOLD:ACN6664	*Phora pubipes*	Joroinen	62.306	27.706	27-Jun-2021	WiT
ZMUO.068329	BOLD:AEB8779	*Phora convergens*	Joroinen	62.306	27.706	27-Jun-2021	WiT
ZMUO.068332	BOLD:ACN6664	*Phora pubipes*	Joroinen	62.306	27.706	27-Jun-2021	WiT
ZMUO.068338	BOLD:ABA7002	*Phora tincta*	Joroinen	62.306	27.706	27-Jun-2021	WiT
ZMUO.068341	BOLD:ACN6664	*Phora pubipes*	Joroinen	62.304	27.71	27-Jun-2021	WiT
ZMUO.068344	BOLD:AEB8779	*Phora convergens*	Joroinen	62.304	27.71	27-Jun-2021	WiT
ZMUO.068346	BOLD:ACI8210	*Phora convergens*	Joroinen	62.304	27.71	27-Jun-2021	WiT
ZMUO.068350	BOLD:AEB8779	*Phora convergens*	Joroinen	62.304	27.71	27-Jun-2021	WiT
ZMUO.068352	BOLD:ABA7002	*Phora tincta*	Joroinen	62.304	27.71	27-Jun-2021	WiT
ZMUO.068353	BOLD:ACI8210	*Phora convergens*	Joroinen	62.304	27.71	27-Jun-2021	WiT
ZMUO.068359	BOLD:ACN6664	*Phora pubipes*	Joroinen	62.304	27.71	27-Jun-2021	WiT
ZMUO.068363	BOLD:ABA7002	*Phora tincta*	Joroinen	62.304	27.71	27-Jun-2021	WiT
ZMUO.068367	BOLD:ACN6664	*Phora pubipes*	Joroinen	62.304	27.71	27-Jun-2021	WiT
ZMUO.068374	BOLD:ABA7002	*Phora tincta*	Joroinen	62.304	27.71	27-Jun-2021	WiT
ZMUO.068376	BOLD:ACN6664	*Phora pubipes*	Joroinen	62.304	27.71	27-Jun-2021	WiT
ZMUO.068383	BOLD:ACN6664	*Phora pubipes*	Joroinen	62.304	27.71	27-Jun-2021	WiT
ZMUO.068387	BOLD:ACN6664	*Phora pubipes*	Joroinen	62.304	27.71	27-Jun-2021	WiT
ZMUO.068388	BOLD:ACN6664	*Phora pubipes*	Joroinen	62.304	27.71	27-Jun-2021	WiT
ZMUO.068395	BOLD:ACN6664	*Phora pubipes*	Joroinen	62.304	27.71	27-Jun-2021	WiT
ZMUO.068399	BOLD:ACN6664	*Phora pubipes*	Joroinen	62.304	27.71	27-Jun-2021	WiT
ZMUO.068402	BOLD:ABA7002	*Phora tincta*	Joroinen	62.304	27.71	27-Jun-2021	WiT
ZMUO.068403	BOLD:ACN6664	*Phora pubipes*	Joroinen	62.304	27.71	27-Jun-2021	WiT
ZMUO.068405	BOLD:ABA7002	*Phora tincta*	Joroinen	62.304	27.71	27-Jun-2021	WiT
ZMUO.068411	BOLD:ACN6664	*Phora pubipes*	Joroinen	62.304	27.71	27-Jun-2021	WiT
ZMUO.068415	BOLD:ACN6664	*Phora pubipes*	Joroinen	62.304	27.71	27-Jun-2021	WiT
ZMUO.068418	BOLD:ACN6664	*Phora pubipes*	Joroinen	62.304	27.71	27-Jun-2021	WiT
ZMUO.068428	BOLD:ACN6664	*Phora pubipes*	Joroinen	62.304	27.71	27-Jun-2021	WiT
ZMUO.068434	BOLD:ACN6664	*Phora pubipes*	Joroinen	62.304	27.71	27-Jun-2021	WiT
ZMUO.068435	BOLD:ABA7002	*Phora tincta*	Joroinen	62.304	27.71	27-Jun-2021	WiT
ZMUO.068439	BOLD:ACN6664	*Phora pubipes*	Joroinen	62.304	27.71	27-Jun-2021	WiT
ZMUO.068450	BOLD:ACI8210	*Phora convergens*	Joroinen	62.304	27.71	27-Jun-2021	WiT
ZMUO.068451	BOLD:ACN6664	*Phora pubipes*	Joroinen	62.304	27.71	27-Jun-2021	WiT
ZMUO.068458	BOLD:ACN6664	*Phora pubipes*	Joroinen	62.304	27.71	27-Jun-2021	WiT
ZMUO.068462	BOLD:ACN6664	*Phora pubipes*	Joroinen	62.304	27.71	27-Jun-2021	WiT
ZMUO.068471	BOLD:ACN6664	*Phora pubipes*	Joroinen	62.304	27.71	27-Jun-2021	WiT
ZMUO.068480	BOLD:AEB8779	*Phora convergens*	Joroinen	62.304	27.71	27-Jun-2021	WiT
ZMUO.068482	BOLD:ACI8210	*Phora convergens*	Joroinen	62.304	27.71	27-Jun-2021	WiT
ZMUO.068487	BOLD:ACN6664	*Phora pubipes*	Joroinen	62.304	27.71	27-Jun-2021	WiT
ZMUO.068488	BOLD:ACN6664	*Phora pubipes*	Joroinen	62.304	27.71	27-Jun-2021	WiT
ZMUO.068501	BOLD:ACN6664	*Phora pubipes*	Joroinen	62.304	27.709	27-Jun-2021	WiT
ZMUO.068502	BOLD:ACN6664	*Phora pubipes*	Joroinen	62.304	27.709	27-Jun-2021	WiT
ZMUO.068504	BOLD:ACN6664	*Phora pubipes*	Joroinen	62.304	27.709	27-Jun-2021	WiT
ZMUO.068505	BOLD:AEB8779	*Phora convergens*	Joroinen	62.304	27.709	27-Jun-2021	WiT
ZMUO.068521	BOLD:ACN6664	*Phora pubipes*	Joroinen	62.304	27.709	27-Jun-2021	WiT
ZMUO.068528	BOLD:ACN6664	*Phora pubipes*	Joroinen	62.304	27.709	27-Jun-2021	WiT
ZMUO.068533	BOLD:AEB8779	*Phora convergens*	Joroinen	62.304	27.709	27-Jun-2021	WiT
ZMUO.068535	BOLD:ACN6664	*Phora pubipes*	Joroinen	62.304	27.709	27-Jun-2021	WiT
ZMUO.068536	BOLD:ABA7002	*Phora tincta*	Joroinen	62.304	27.709	27-Jun-2021	WiT

ZMUO.068540	BOLD:ACN6664	*Phora pubipes*	Joroinen	62.304	27.709	27-Jun-2021	WiT
ZMUO.068542	BOLD:ACN6664	*Phora pubipes*	Joroinen	62.304	27.709	27-Jun-2021	WiT
ZMUO.068546	BOLD:ACN6664	*Phora pubipes*	Joroinen	62.304	27.709	27-Jun-2021	WiT
ZMUO.068553	BOLD:ABA7002	*Phora tincta*	Joroinen	62.304	27.709	27-Jun-2021	WiT
ZMUO.068554	BOLD:ACN6664	*Phora pubipes*	Joroinen	62.304	27.709	27-Jun-2021	WiT
ZMUO.068558	BOLD:ACN6664	*Phora pubipes*	Joroinen	62.304	27.709	27-Jun-2021	WiT
ZMUO.068567	BOLD:ACN6664	*Phora pubipes*	Joroinen	62.304	27.709	27-Jun-2021	WiT
ZMUO.068568	BOLD:ACN6664	*Phora pubipes*	Joroinen	62.304	27.709	27-Jun-2021	WiT
ZMUO.068571	BOLD:ACN6664	*Phora pubipes*	Joroinen	62.304	27.709	27-Jun-2021	WiT
ZMUO.068573	BOLD:ACN6664	*Phora pubipes*	Joroinen	62.304	27.709	27-Jun-2021	WiT
ZMUO.068574	BOLD:ABA7002	*Phora tincta*	Joroinen	62.304	27.709	27-Jun-2021	WiT
ZMUO.068576	BOLD:ACN6664	*Phora pubipes*	Joroinen	62.304	27.709	27-Jun-2021	WiT
ZMUO.068582	BOLD:ACN6664	*Phora pubipes*	Joroinen	62.304	27.709	27-Jun-2021	WiT
ZMUO.068583	BOLD:ACN6664	*Phora pubipes*	Joroinen	62.304	27.709	27-Jun-2021	WiT
ZMUO.068592	BOLD:AEB8779	*Phora convergens*	Joroinen	62.304	27.709	27-Jun-2021	WiT
ZMUO.068593	BOLD:ACN6664	*Phora pubipes*	Joroinen	62.304	27.709	27-Jun-2021	WiT
ZMUO.068597	BOLD:ACN6664	*Phora pubipes*	Joroinen	62.304	27.709	27-Jun-2021	WiT
ZMUO.068599	BOLD:ACN6664	*Phora pubipes*	Joroinen	62.304	27.709	27-Jun-2021	WiT
ZMUO.068601	BOLD:ACN6664	*Phora pubipes*	Joroinen	62.304	27.709	27-Jun-2021	WiT
ZMUO.068605	BOLD:ABA7002	*Phora tincta*	Joroinen	62.304	27.709	27-Jun-2021	WiT
ZMUO.068614	BOLD:AEB8779	*Phora convergens*	Joroinen	62.304	27.709	27-Jun-2021	WiT
ZMUO.068625	BOLD:ABA7002	*Phora tincta*	Joroinen	62.304	27.709	27-Jun-2021	WiT
ZMUO.068626	BOLD:ABA7002	*Phora tincta*	Joroinen	62.304	27.709	27-Jun-2021	WiT
ZMUO.068631	BOLD:ACN6664	*Phora pubipes*	Joroinen	62.304	27.709	27-Jun-2021	WiT
ZMUO.068637	BOLD:ACN6664	*Phora pubipes*	Joroinen	62.304	27.709	27-Jun-2021	WiT
ZMUO.068639	BOLD:ACN6664	*Phora pubipes*	Joroinen	62.304	27.709	27-Jun-2021	WiT
ZMUO.068640	BOLD:ACN6664	*Phora pubipes*	Joroinen	62.304	27.709	27-Jun-2021	WiT
ZMUO.068646	BOLD:ACN6664	*Phora pubipes*	Joroinen	62.304	27.709	27-Jun-2021	WiT
ZMUO.068651	BOLD:AEB8779	*Phora convergens*	Joroinen	62.304	27.709	27-Jun-2021	WiT
ZMUO.068653	BOLD:ACQ9655	*Anevrina unispinosa*	Joroinen	62.304	27.709	27-Jun-2021	WiT
ZMUO.068658	BOLD:ACN6664	*Phora pubipes*	Joroinen	62.304	27.709	27-Jun-2021	WiT
ZMUO.068669	BOLD:ACN6664	*Phora pubipes*	Joroinen	62.304	27.709	27-Jun-2021	WiT
ZMUO.068670	BOLD:ACN6664	*Phora pubipes*	Joroinen	62.304	27.709	27-Jun-2021	WiT
ZMUO.068680	BOLD:ACN6664	*Phora pubipes*	Joroinen	62.304	27.709	27-Jun-2021	WiT
ZMUO.068684	BOLD:ACN6664	*Phora pubipes*	Joroinen	62.304	27.709	27-Jun-2021	WiT
ZMUO.068687	BOLD:AEB8779	*Phora convergens*	Joroinen	62.3	27.701	27-Jun-2021	WiT
ZMUO.068688	BOLD:ABA7002	*Phora tincta*	Joroinen	62.3	27.701	27-Jun-2021	WiT
ZMUO.068692	BOLD:AEB8779	*Phora convergens*	Joroinen	62.3	27.701	27-Jun-2021	WiT
ZMUO.068695	BOLD:ABA7002	*Phora tincta*	Joroinen	62.3	27.701	27-Jun-2021	WiT
ZMUO.068700	BOLD:ACI8210	*Phora convergens*	Joroinen	62.3	27.701	27-Jun-2021	WiT
ZMUO.068707	BOLD:AEB8779	*Phora convergens*	Joroinen	62.3	27.701	27-Jun-2021	WiT
ZMUO.068711	BOLD:AEB8779	*Phora convergens*	Joroinen	62.3	27.701	27-Jun-2021	WiT
ZMUO.068712	BOLD:ABA7002	*Phora tincta*	Joroinen	62.3	27.701	27-Jun-2021	WiT
ZMUO.068714	BOLD:ACN6664	*Phora pubipes*	Joroinen	62.3	27.701	27-Jun-2021	WiT
ZMUO.068729	BOLD:AEB8779	*Phora convergens*	Joroinen	62.3	27.701	27-Jun-2021	WiT
ZMUO.068743	BOLD:ACI8210	*Phora convergens*	Joroinen	62.3	27.701	27-Jun-2021	WiT
ZMUO.068744	BOLD:AFE7212	*Phora holosericea*	Joroinen	62.3	27.701	27-Jun-2021	WiT
ZMUO.068746	BOLD:ACN6664	*Phora pubipes*	Joroinen	62.3	27.701	27-Jun-2021	WiT

ZMUO.068748	BOLD:ABA7002	*Phora tincta*	Joroinen	62.3	27.701	27-Jun-2021	WiT
ZMUO.068756	BOLD:ACN6664	*Phora pubipes*	Joroinen	62.3	27.701	27-Jun-2021	WiT
ZMUO.068757	BOLD:ABA7002	*Phora tincta*	Joroinen	62.3	27.701	27-Jun-2021	WiT
ZMUO.068760	BOLD:ACN6664	*Phora pubipes*	Joroinen	62.3	27.701	27-Jun-2021	WiT
ZMUO.068763	BOLD:ACN6664	*Phora pubipes*	Joroinen	62.3	27.701	27-Jun-2021	WiT
ZMUO.068764	BOLD:ABA7002	*Phora tincta*	Joroinen	62.3	27.701	27-Jun-2021	WiT
ZMUO.068766	BOLD:AEB8779	*Phora convergens*	Joroinen	62.3	27.701	27-Jun-2021	WiT
ZMUO.068769	BOLD:ACN6664	*Phora pubipes*	Joroinen	62.3	27.701	27-Jun-2021	WiT
ZMUO.068774	BOLD:AEB8779	*Phora convergens*	Joroinen	62.3	27.701	27-Jun-2021	WiT
ZMUO.068777	BOLD:ABA7002	*Phora tincta*	Joroinen	62.3	27.701	27-Jun-2021	WiT
ZMUO.068780	BOLD:ACN6664	*Phora pubipes*	Joroinen	62.3	27.701	27-Jun-2021	WiT
ZMUO.068784	BOLD:ACN6664	*Phora pubipes*	Joroinen	62.3	27.701	27-Jun-2021	WiT
ZMUO.068787	BOLD:AEB8779	*Phora convergens*	Joroinen	62.3	27.701	27-Jun-2021	WiT
ZMUO.068788	BOLD:AEB8779	*Phora convergens*	Joroinen	62.3	27.701	27-Jun-2021	WiT
ZMUO.068789	BOLD:AEB8779	*Phora convergens*	Joroinen	62.3	27.701	27-Jun-2021	WiT
ZMUO.068792	BOLD:ACN6664	*Phora pubipes*	Joroinen	62.3	27.701	27-Jun-2021	WiT
ZMUO.068793	BOLD:ACN6664	*Phora pubipes*	Joroinen	62.3	27.701	27-Jun-2021	WiT
ZMUO.068796	BOLD:ACN6664	*Phora pubipes*	Joroinen	62.3	27.701	27-Jun-2021	WiT
ZMUO.068800	BOLD:ABA7002	*Phora tincta*	Joroinen	62.3	27.701	27-Jun-2021	WiT
ZMUO.068802	BOLD:ACN6664	*Phora pubipes*	Joroinen	62.3	27.701	27-Jun-2021	WiT
ZMUO.068804	BOLD:ABA7002	*Phora tincta*	Joroinen	62.3	27.701	27-Jun-2021	WiT
ZMUO.068805	BOLD:ACN6664	*Phora pubipes*	Joroinen	62.3	27.701	27-Jun-2021	WiT
ZMUO.068807	BOLD:ACN6664	*Phora pubipes*	Joroinen	62.3	27.701	27-Jun-2021	WiT
ZMUO.068808	BOLD:ACN6664	*Phora pubipes*	Joroinen	62.3	27.701	27-Jun-2021	WiT
ZMUO.068810	BOLD:AEB8779	*Phora convergens*	Joroinen	62.3	27.701	27-Jun-2021	WiT
ZMUO.068812	BOLD:ABA7002	*Phora tincta*	Joroinen	62.3	27.701	27-Jun-2021	WiT
ZMUO.068813	BOLD:AEB8779	*Phora convergens*	Joroinen	62.3	27.701	27-Jun-2021	WiT
ZMUO.068814	BOLD:ABA7002	*Phora tincta*	Joroinen	62.3	27.701	27-Jun-2021	WiT
ZMUO.068819	BOLD:ABA7002	*Phora tincta*	Joroinen	62.3	27.701	27-Jun-2021	WiT
ZMUO.068821	BOLD:ACN6664	*Phora pubipes*	Joroinen	62.3	27.701	27-Jun-2021	WiT
ZMUO.068822	BOLD:ACN6664	*Phora pubipes*	Joroinen	62.3	27.701	27-Jun-2021	WiT
ZMUO.068823	BOLD:AEB8779	*Phora convergens*	Joroinen	62.3	27.701	27-Jun-2021	WiT
ZMUO.068827	BOLD:AEB8779	*Phora convergens*	Joroinen	62.3	27.701	27-Jun-2021	WiT
ZMUO.068829	BOLD:ACN6664	*Phora pubipes*	Joroinen	62.3	27.701	27-Jun-2021	WiT
ZMUO.068832	BOLD:ABA7002	*Phora tincta*	Joroinen	62.3	27.701	27-Jun-2021	WiT
ZMUO.068834	BOLD:ACN6664	*Phora pubipes*	Joroinen	62.3	27.701	27-Jun-2021	WiT
ZMUO.068837	BOLD:ABA7002	*Phora tincta*	Joroinen	62.3	27.701	27-Jun-2021	WiT
ZMUO.068841	BOLD:ACN6664	*Phora pubipes*	Joroinen	62.3	27.701	27-Jun-2021	WiT
ZMUO.068843	BOLD:ACN6664	*Phora pubipes*	Joroinen	62.3	27.701	27-Jun-2021	WiT
ZMUO.068844	BOLD:ACN6664	*Phora pubipes*	Joroinen	62.3	27.701	27-Jun-2021	WiT
ZMUO.068853	BOLD:ACN6664	*Phora pubipes*	Joroinen	62.3	27.701	27-Jun-2021	WiT
ZMUO.068855	BOLD:ACN6664	*Phora pubipes*	Joroinen	62.3	27.701	27-Jun-2021	WiT
ZMUO.068856	BOLD:ACN6664	*Phora pubipes*	Joroinen	62.3	27.701	27-Jun-2021	WiT
ZMUO.068857	BOLD:ABA7002	*Phora tincta*	Joroinen	62.3	27.701	27-Jun-2021	WiT
ZMUO.068860	BOLD:ACN6664	*Phora pubipes*	Joroinen	62.3	27.701	27-Jun-2021	WiT
ZMUO.068861	BOLD:ABA7002	*Phora tincta*	Joroinen	62.3	27.701	27-Jun-2021	WiT
ZMUO.068866	BOLD:ABA7002	*Phora tincta*	Joroinen	62.3	27.701	27-Jun-2021	WiT
ZMUO.068868	BOLD:ACN6664	*Phora pubipes*	Joroinen	62.3	27.701	27-Jun-2021	WiT

ZMUO.068869	BOLD:ABA7002	*Phora tincta*	Joroinen	62.3	27.701	27-Jun-2021	WiT
ZMUO.068870	BOLD:AEB8779	*Phora convergens*	Joroinen	62.3	27.701	27-Jun-2021	WiT
ZMUO.068874	BOLD:ACN6664	*Phora pubipes*	Joroinen	62.3	27.701	27-Jun-2021	WiT
ZMUO.068875	BOLD:ACN6664	*Phora pubipes*	Joroinen	62.3	27.701	27-Jun-2021	WiT
ZMUO.068877	BOLD:ABA7002	*Phora tincta*	Joroinen	62.3	27.701	27-Jun-2021	WiT
ZMUO.068881	BOLD:ABA7002	*Phora tincta*	Joroinen	62.3	27.701	27-Jun-2021	WiT
ZMUO.068882	BOLD:ABA7002	*Phora tincta*	Joroinen	62.3	27.701	27-Jun-2021	WiT
ZMUO.068883	BOLD:AEB8779	*Phora convergens*	Joroinen	62.3	27.701	27-Jun-2021	WiT
ZMUO.068884	BOLD:ACN6664	*Phora pubipes*	Joroinen	62.3	27.701	27-Jun-2021	WiT
ZMUO.068885	BOLD:ABA7002	*Phora tincta*	Joroinen	62.3	27.701	27-Jun-2021	WiT
ZMUO.068886	BOLD:ACN6664	*Phora pubipes*	Joroinen	62.3	27.701	27-Jun-2021	WiT
ZMUO.068889	BOLD:ACB4872	*Phora atra*	Joroinen	62.3	27.701	27-Jun-2021	WiT
ZMUO.068890	BOLD:AEB8779	*Phora convergens*	Joroinen	62.3	27.701	27-Jun-2021	WiT
ZMUO.068895	BOLD:AEB8779	*Phora convergens*	Joroinen	62.3	27.701	27-Jun-2021	WiT
ZMUO.068897	BOLD:ACN6664	*Phora pubipes*	Joroinen	62.3	27.701	27-Jun-2021	WiT
ZMUO.068898	BOLD:ABA7002	*Phora tincta*	Joroinen	62.3	27.701	27-Jun-2021	WiT
ZMUO.068901	BOLD:ACN6664	*Phora pubipes*	Joroinen	62.3	27.701	27-Jun-2021	WiT
ZMUO.068905	BOLD:ABA7002	*Phora tincta*	Joroinen	62.3	27.701	27-Jun-2021	WiT
ZMUO.068907	BOLD:ABA7002	*Phora tincta*	Joroinen	62.3	27.701	27-Jun-2021	WiT
ZMUO.068912	BOLD:ABA7002	*Phora tincta*	Joroinen	62.3	27.701	27-Jun-2021	WiT
ZMUO.068913	BOLD:ABA7002	*Phora tincta*	Joroinen	62.3	27.701	27-Jun-2021	WiT
ZMUO.068917	BOLD:ACN6664	*Phora pubipes*	Joroinen	62.3	27.701	27-Jun-2021	WiT
ZMUO.068919	BOLD:ABA7002	*Phora tincta*	Joroinen	62.3	27.701	27-Jun-2021	WiT
ZMUO.068921	BOLD:ACN6664	*Phora pubipes*	Joroinen	62.3	27.701	27-Jun-2021	WiT
ZMUO.068922	BOLD:ACN6664	*Phora pubipes*	Joroinen	62.3	27.701	27-Jun-2021	WiT
ZMUO.068923	BOLD:ABA7002	*Phora tincta*	Joroinen	62.3	27.701	27-Jun-2021	WiT
ZMUO.068924	BOLD:ACN6664	*Phora pubipes*	Joroinen	62.3	27.701	27-Jun-2021	WiT
ZMUO.068926	BOLD:AEB8779	*Phora convergens*	Joroinen	62.3	27.701	27-Jun-2021	WiT
ZMUO.068929	BOLD:AEB8779	*Phora convergens*	Joroinen	62.3	27.701	27-Jun-2021	WiT
ZMUO.068932	BOLD:ABA7002	*Phora tincta*	Joroinen	62.3	27.701	27-Jun-2021	WiT
ZMUO.068933	BOLD:ACN6664	*Phora pubipes*	Joroinen	62.3	27.701	27-Jun-2021	WiT
ZMUO.068934	BOLD:ACN6664	*Phora pubipes*	Joroinen	62.3	27.701	27-Jun-2021	WiT
ZMUO.068935	BOLD:ACN6664	*Phora pubipes*	Joroinen	62.3	27.701	27-Jun-2021	WiT
ZMUO.068936	BOLD:ACN6664	*Phora pubipes*	Joroinen	62.3	27.701	27-Jun-2021	WiT
ZMUO.068937	BOLD:ACN6664	*Phora pubipes*	Joroinen	62.3	27.701	27-Jun-2021	WiT
ZMUO.068938	BOLD:ABA7002	*Phora tincta*	Joroinen	62.3	27.701	27-Jun-2021	WiT
ZMUO.068945	BOLD:ABA7002	*Phora tincta*	Joroinen	62.3	27.701	27-Jun-2021	WiT
ZMUO.068949	BOLD:ABA7002	*Phora tincta*	Joroinen	62.3	27.701	27-Jun-2021	WiT
ZMUO.069039	BOLD:ADM5152	*Triphleba antricola*	Inkoo	60.069	23.863	13-Jul-2020	MaT
ZMUO.069053	BOLD:ACB3701	*Diplonevra concinna*	Inkoo	60.069	23.863	22-Aug-2020	MaT
ZMUO.069322	BOLD:ABA7001	*Diplonevra glabra*	Kristiinankaupunki	62.282	21.409	23-Jun-2020	MaT
ZMUO.069328	BOLD:ABA7001	*Diplonevra glabra*	Kristiinankaupunki	62.282	21.409	23-Jun-2020	MaT
ZMUO.069338	BOLD:ABA7001	*Diplonevra glabra*	Kristiinankaupunki	62.282	21.409	23-Jun-2020	MaT
ZMUO.069345	BOLD:ABA7001	*Diplonevra glabra*	Kristiinankaupunki	62.282	21.409	23-Jun-2020	MaT
ZMUO.069359	BOLD:ACE2645	*Conicera floricola*	Kristiinankaupunki	62.282	21.409	23-Jun-2020	MaT
ZMUO.069385	BOLD:ADM8144	*Phora obscura*	Kristiinankaupunki	62.282	21.409	23-Jun-2020	MaT
ZMUO.069392	BOLD:ABA7002	*Phora tincta*	Kristiinankaupunki	62.282	21.409	23-Jun-2020	MaT
ZMUO.069412	BOLD:AAG3236	*Diplonevra nitidula*	Kristiinankaupunki	62.282	21.409	23-Jun-2020	MaT

ZMUO.069423	BOLD:ABA7002	*Phora tincta*	Kristiinankaupunki	62.282	21.409	23-Jun-2020	MaT
ZMUO.069441	BOLD:ACO9836	*Triphleba distinguenda*	Kristiinankaupunki	62.282	21.409	14-Jul-2020	MaT
ZMUO.069468	BOLD:AAG3236	*Diplonevra nitidula*	Kristiinankaupunki	62.282	21.409	14-Jul-2020	MaT
ZMUO.069485	BOLD:ACO9836	*Triphleba distinguenda*	Kristiinankaupunki	62.282	21.409	14-Jul-2020	MaT
ZMUO.069486	BOLD:ACO9836	*Triphleba distinguenda*	Kristiinankaupunki	62.282	21.409	14-Jul-2020	MaT
ZMUO.069501	BOLD:ACB4872	*Phora atra*	Kristiinankaupunki	62.282	21.409	14-Jul-2020	MaT
ZMUO.069515	BOLD:AAG3236	*Diplonevra nitidula*	Kristiinankaupunki	62.282	21.409	14-Jul-2020	MaT
ZMUO.069516	BOLD:ACO9836	*Triphleba distinguenda*	Kristiinankaupunki	62.282	21.409	14-Jul-2020	MaT
ZMUO.069528	BOLD:ADM5152	*Triphleba antricola*	Kristiinankaupunki	62.282	21.409	14-Jul-2020	MaT
ZMUO.069536	BOLD:AAG3236	*Diplonevra nitidula*	Kristiinankaupunki	62.282	21.409	14-Jul-2020	MaT
ZMUO.069558	BOLD:ACO9836	*Triphleba distinguenda*	Kristiinankaupunki	62.282	21.409	14-Jul-2020	MaT
ZMUO.069560	BOLD:ACO9836	*Triphleba distinguenda*	Kristiinankaupunki	62.282	21.409	04-Aug-2020	MaT
ZMUO.069564	BOLD:ACO9836	*Triphleba distinguenda*	Kristiinankaupunki	62.282	21.409	04-Aug-2020	MaT
ZMUO.069565	BOLD:ACO9836	*Triphleba distinguenda*	Kristiinankaupunki	62.282	21.409	04-Aug-2020	MaT
ZMUO.069568	BOLD:ADM5152	*Triphleba antricola*	Kristiinankaupunki	62.282	21.409	04-Aug-2020	MaT
ZMUO.069570	BOLD:ACO9836	*Triphleba distinguenda*	Kristiinankaupunki	62.282	21.409	04-Aug-2020	MaT
ZMUO.069571	BOLD:ACO9836	*Triphleba distinguenda*	Kristiinankaupunki	62.282	21.409	04-Aug-2020	MaT
ZMUO.069579	BOLD:ACD2464	*Borophaga carinifrons*	Kristiinankaupunki	62.282	21.409	04-Aug-2020	MaT
ZMUO.069580	BOLD:ACD2464	*Borophaga carinifrons*	Kristiinankaupunki	62.282	21.409	04-Aug-2020	MaT
ZMUO.069585	BOLD:ACO9836	*Triphleba distinguenda*	Kristiinankaupunki	62.282	21.409	04-Aug-2020	MaT
ZMUO.069586	BOLD:ACO9836	*Triphleba distinguenda*	Kristiinankaupunki	62.282	21.409	04-Aug-2020	MaT
ZMUO.069587	BOLD:ACD2464	*Borophaga carinifrons*	Kristiinankaupunki	62.282	21.409	04-Aug-2020	MaT
ZMUO.069588	BOLD:ACD2464	*Borophaga carinifrons*	Kristiinankaupunki	62.282	21.409	04-Aug-2020	MaT
ZMUO.069589	BOLD:ADM5152	*Triphleba antricola*	Kristiinankaupunki	62.282	21.409	04-Aug-2020	MaT
ZMUO.069590	BOLD:ACO9836	*Triphleba distinguenda*	Kristiinankaupunki	62.282	21.409	04-Aug-2020	MaT
ZMUO.069595	BOLD:ACD2464	*Borophaga carinifrons*	Kristiinankaupunki	62.282	21.409	04-Aug-2020	MaT
ZMUO.069597	BOLD:ACO9836	*Triphleba distinguenda*	Kristiinankaupunki	62.282	21.409	04-Aug-2020	MaT
ZMUO.069603	BOLD:ACO9836	*Triphleba distinguenda*	Kristiinankaupunki	62.282	21.409	04-Aug-2020	MaT
ZMUO.069606	BOLD:ADM5152	*Triphleba antricola*	Kristiinankaupunki	62.282	21.409	04-Aug-2020	MaT
ZMUO.069611	BOLD:ACO9836	*Triphleba distinguenda*	Kristiinankaupunki	62.282	21.409	04-Aug-2020	MaT
ZMUO.069613	BOLD:ACO9836	*Triphleba distinguenda*	Kristiinankaupunki	62.282	21.409	04-Aug-2020	MaT
ZMUO.069615	BOLD:ACD2464	*Borophaga carinifrons*	Kristiinankaupunki	62.282	21.409	04-Aug-2020	MaT
ZMUO.069621	BOLD:ACD2464	*Borophaga carinifrons*	Kristiinankaupunki	62.282	21.409	04-Aug-2020	MaT
ZMUO.069623	BOLD:ACO9836	*Triphleba distinguenda*	Kristiinankaupunki	62.282	21.409	04-Aug-2020	MaT
ZMUO.069624	BOLD:ACO9836	*Triphleba distinguenda*	Kristiinankaupunki	62.282	21.409	04-Aug-2020	MaT
ZMUO.069627	BOLD:ACO9836	*Triphleba distinguenda*	Kristiinankaupunki	62.282	21.409	04-Aug-2020	MaT
ZMUO.069629	BOLD:ACO9836	*Triphleba distinguenda*	Kristiinankaupunki	62.282	21.409	04-Aug-2020	MaT
ZMUO.069630	BOLD:ACB3701	*Diplonevra concinna*	Kristiinankaupunki	62.282	21.409	04-Aug-2020	MaT
ZMUO.069638	BOLD:ACO9836	*Triphleba distinguenda*	Kristiinankaupunki	62.282	21.409	04-Aug-2020	MaT
ZMUO.069652	BOLD:ACO9836	*Triphleba distinguenda*	Kristiinankaupunki	62.282	21.409	04-Aug-2020	MaT
ZMUO.069653	BOLD:ACO9836	*Triphleba distinguenda*	Kristiinankaupunki	62.282	21.409	04-Aug-2020	MaT
ZMUO.069656	BOLD:ACD2464	*Borophaga carinifrons*	Kristiinankaupunki	62.282	21.409	23-Aug-2020	MaT
ZMUO.069657	BOLD:ACD2464	*Borophaga carinifrons*	Kristiinankaupunki	62.282	21.409	23-Aug-2020	MaT
ZMUO.069658	BOLD:ACD2464	*Borophaga carinifrons*	Kristiinankaupunki	62.282	21.409	23-Aug-2020	MaT
ZMUO.069659	BOLD:ACD2464	*Borophaga carinifrons*	Kristiinankaupunki	62.282	21.409	23-Aug-2020	MaT
ZMUO.069662	BOLD:ACQ3921	*Phora stictica*	Kristiinankaupunki	62.282	21.409	23-Aug-2020	MaT
ZMUO.069663	BOLD:ACD2464	*Borophaga carinifrons*	Kristiinankaupunki	62.282	21.409	23-Aug-2020	MaT
ZMUO.069664	BOLD:ACB3701	*Diplonevra concinna*	Kristiinankaupunki	62.282	21.409	23-Aug-2020	MaT

ZMUO.069665	BOLD:ACD2464	*Borophaga carinifrons*	Kristiinankaupunki	62.282	21.409	23-Aug-2020	MaT
ZMUO.069667	BOLD:ACD2464	*Borophaga carinifrons*	Kristiinankaupunki	62.282	21.409	23-Aug-2020	MaT
ZMUO.069668	BOLD:ACD2464	*Borophaga carinifrons*	Kristiinankaupunki	62.282	21.409	23-Aug-2020	MaT
ZMUO.069669	BOLD:ACD2464	*Borophaga carinifrons*	Kristiinankaupunki	62.282	21.409	23-Aug-2020	MaT
ZMUO.069670	BOLD:ACD2464	*Borophaga carinifrons*	Kristiinankaupunki	62.282	21.409	23-Aug-2020	MaT
ZMUO.069671	BOLD:ACD2464	*Borophaga carinifrons*	Kristiinankaupunki	62.282	21.409	23-Aug-2020	MaT
ZMUO.069672	BOLD:ACD2464	*Borophaga carinifrons*	Kristiinankaupunki	62.282	21.409	23-Aug-2020	MaT
ZMUO.069674	BOLD:ACD2464	*Borophaga carinifrons*	Kristiinankaupunki	62.282	21.409	23-Aug-2020	MaT
ZMUO.069678	BOLD:ACD2464	*Borophaga carinifrons*	Kristiinankaupunki	62.282	21.409	23-Aug-2020	MaT
ZMUO.069679	BOLD:ACD2464	*Borophaga carinifrons*	Kristiinankaupunki	62.282	21.409	23-Aug-2020	MaT
ZMUO.069681	BOLD:ACO9836	*Triphleba distinguenda*	Kristiinankaupunki	62.282	21.409	23-Aug-2020	MaT
ZMUO.069683	BOLD:ACD2464	*Borophaga carinifrons*	Kristiinankaupunki	62.282	21.409	23-Aug-2020	MaT
ZMUO.069685	BOLD:ACD2464	*Borophaga carinifrons*	Kristiinankaupunki	62.282	21.409	23-Aug-2020	MaT
ZMUO.069686	BOLD:ACD2464	*Borophaga carinifrons*	Kristiinankaupunki	62.282	21.409	23-Aug-2020	MaT
ZMUO.069687	BOLD:ACD2464	*Borophaga carinifrons*	Kristiinankaupunki	62.282	21.409	23-Aug-2020	MaT
ZMUO.069688	BOLD:ACO9836	*Triphleba distinguenda*	Kristiinankaupunki	62.282	21.409	23-Aug-2020	MaT
ZMUO.069689	BOLD:ACD2464	*Borophaga carinifrons*	Kristiinankaupunki	62.282	21.409	23-Aug-2020	MaT
ZMUO.069690	BOLD:ACD2464	*Borophaga carinifrons*	Kristiinankaupunki	62.282	21.409	23-Aug-2020	MaT
ZMUO.069691	BOLD:ACD2464	*Borophaga carinifrons*	Kristiinankaupunki	62.282	21.409	23-Aug-2020	MaT
ZMUO.069692	BOLD:ADM5152	*Triphleba antricola*	Kristiinankaupunki	62.282	21.409	23-Aug-2020	MaT
ZMUO.069696	BOLD:ACD2464	*Borophaga carinifrons*	Kristiinankaupunki	62.282	21.409	23-Aug-2020	MaT
ZMUO.069697	BOLD:ACB3701	*Diplonevra concinna*	Kristiinankaupunki	62.282	21.409	23-Aug-2020	MaT
ZMUO.069703	BOLD:ABA7002	*Phora tincta*	Turku	60.432	22.162	22-Jun-2020	MaT
ZMUO.069704	BOLD:ABA7002	*Phora tincta*	Turku	60.432	22.162	22-Jun-2020	MaT
ZMUO.069716	BOLD:ABA7002	*Phora tincta*	Turku	60.432	22.162	22-Jun-2020	MaT
ZMUO.069719	BOLD:ABA7002	*Phora tincta*	Turku	60.432	22.162	22-Jun-2020	MaT
ZMUO.069721	BOLD:ADM5152	*Triphleba antricola*	Turku	60.432	22.162	22-Jun-2020	MaT
ZMUO.069733	BOLD:ADM5152	*Triphleba antricola*	Turku	60.432	22.162	22-Jun-2020	MaT
ZMUO.069735	BOLD:ABA7001	*Diplonevra glabra*	Turku	60.432	22.162	22-Jun-2020	MaT
ZMUO.069784	BOLD:ADM5152	*Triphleba antricola*	Turku	60.432	22.162	22-Jun-2020	MaT
ZMUO.069787	BOLD:ACD2261	*Phora artifrons*	Turku	60.432	22.162	22-Jun-2020	MaT
ZMUO.069792	BOLD:ADM5152	*Triphleba antricola*	Turku	60.432	22.162	22-Jun-2020	MaT
ZMUO.069839	BOLD:ABA7002	*Phora tincta*	Turku	60.432	22.162	22-Jun-2020	MaT
ZMUO.069860	BOLD:ABA7002	*Phora tincta*	Turku	60.432	22.162	22-Jun-2020	MaT
ZMUO.069900	BOLD:ABA7002	*Phora tincta*	Turku	60.432	22.162	14-Jul-2020	MaT
ZMUO.069914	BOLD:ADM5152	*Triphleba antricola*	Turku	60.432	22.162	14-Jul-2020	MaT
ZMUO.069952	BOLD:ADM5152	*Triphleba antricola*	Turku	60.432	22.162	14-Jul-2020	MaT
ZMUO.069978	BOLD:ADM5152	*Triphleba antricola*	Turku	60.432	22.162	14-Jul-2020	MaT
ZMUO.069990	BOLD:ADM5152	*Triphleba antricola*	Turku	60.432	22.162	14-Jul-2020	MaT
ZMUO.070003	BOLD:AAP9864	*Triphleba subcompleta*	Turku	60.432	22.162	14-Jul-2020	MaT
ZMUO.070022	BOLD:ADM5152	*Triphleba antricola*	Turku	60.432	22.162	14-Jul-2020	MaT
ZMUO.070028	BOLD:AAP9864	*Triphleba subcompleta*	Turku	60.432	22.162	14-Jul-2020	MaT
ZMUO.070032	BOLD:ACQ9655	*Anevrina unispinosa*	Turku	60.432	22.162	14-Jul-2020	MaT
ZMUO.070041	BOLD:ACB3401	*Chaetopleurophora erythronota*	Turku	60.432	22.162	14-Jul-2020	MaT
ZMUO.070044	BOLD:ADM5152	*Triphleba antricola*	Turku	60.432	22.162	14-Jul-2020	MaT
ZMUO.070046	BOLD:ADM5152	*Triphleba antricola*	Turku	60.432	22.162	14-Jul-2020	MaT
ZMUO.070050	BOLD:ADM5152	*Triphleba antricola*	Turku	60.432	22.162	14-Jul-2020	MaT
ZMUO.070056	BOLD:ACK2513	*Spiniphora excisa*	Turku	60.432	22.162	14-Jul-2020	MaT

ZMUO.070077	BOLD:ADM5152	*Triphleba antricola*	Turku	60.432	22.162	14-Jul-2020	MaT
ZMUO.070086	BOLD:ADM5152	*Triphleba antricola*	Turku	60.432	22.162	14-Jul-2020	MaT
ZMUO.070101	BOLD:ADM5152	*Triphleba antricola*	Turku	60.432	22.162	04-Aug-2020	MaT
ZMUO.070103	BOLD:ACB3412	*Triphleba nudipalpis*	Turku	60.432	22.162	04-Aug-2020	MaT
ZMUO.070115	BOLD:ACK2513	*Spiniphora excisa*	Turku	60.432	22.162	04-Aug-2020	MaT
ZMUO.070118	BOLD:AAP9864	*Triphleba subcompleta*	Turku	60.432	22.162	04-Aug-2020	MaT
ZMUO.070128	BOLD:ADM5152	*Triphleba antricola*	Turku	60.432	22.162	04-Aug-2020	MaT
ZMUO.070145	BOLD:ADM5152	*Triphleba antricola*	Turku	60.432	22.162	04-Aug-2020	MaT
ZMUO.070146	BOLD:ADM5152	*Triphleba antricola*	Turku	60.432	22.162	04-Aug-2020	MaT
ZMUO.070156	BOLD:ACO9836	*Triphleba distinguenda*	Turku	60.432	22.162	04-Aug-2020	MaT
ZMUO.070173	BOLD:ADM5152	*Triphleba antricola*	Turku	60.432	22.162	04-Aug-2020	MaT
ZMUO.070180	BOLD:ADM5152	*Triphleba antricola*	Turku	60.432	22.162	04-Aug-2020	MaT
ZMUO.070187	BOLD:ADM5152	*Triphleba antricola*	Turku	60.432	22.162	04-Aug-2020	MaT
ZMUO.070188	BOLD:ADM5152	*Triphleba antricola*	Turku	60.432	22.162	04-Aug-2020	MaT
ZMUO.070190	BOLD:AAG3236	*Diplonevra nitidula*	Turku	60.432	22.162	04-Aug-2020	MaT
ZMUO.070202	BOLD:ADM5152	*Triphleba antricola*	Turku	60.432	22.162	04-Aug-2020	MaT
ZMUO.070218	BOLD:ACO9836	*Triphleba distinguenda*	Turku	60.432	22.162	04-Aug-2020	MaT
ZMUO.070223	BOLD:AAG3236	*Diplonevra nitidula*	Turku	60.432	22.162	04-Aug-2020	MaT
ZMUO.070227	BOLD:ABA7002	*Phora tincta*	Turku	60.432	22.162	04-Aug-2020	MaT
ZMUO.070230	BOLD:ADM5152	*Triphleba antricola*	Turku	60.432	22.162	04-Aug-2020	MaT
ZMUO.070231	BOLD:ADM5152	*Triphleba antricola*	Turku	60.432	22.162	04-Aug-2020	MaT
ZMUO.070285	BOLD:ACB3701	*Diplonevra concinna*	Turku	60.432	22.162	23-Aug-2020	MaT
ZMUO.070294	BOLD:ACN6664	*Phora pubipes*	Turku	60.432	22.162	23-Aug-2020	MaT
ZMUO.070298	BOLD:ADM5152	*Triphleba antricola*	Turku	60.432	22.162	23-Aug-2020	MaT
ZMUO.070299	BOLD:ACB3701	*Diplonevra concinna*	Turku	60.432	22.162	23-Aug-2020	MaT
ZMUO.070304	BOLD:ADA0453	*Triphleba citreiformis*	Turku	60.432	22.162	23-Aug-2020	MaT
ZMUO.070308	BOLD:ADM5152	*Triphleba antricola*	Turku	60.432	22.162	23-Aug-2020	MaT
ZMUO.070311	BOLD:ACD2464	*Borophaga carinifrons*	Turku	60.432	22.162	23-Aug-2020	MaT
ZMUO.070316	BOLD:ACB3701	*Diplonevra concinna*	Turku	60.432	22.162	23-Aug-2020	MaT
ZMUO.070329	BOLD:ACB3701	*Diplonevra concinna*	Turku	60.432	22.162	23-Aug-2020	MaT
ZMUO.070335	BOLD:ADM5152	*Triphleba antricola*	Turku	60.432	22.162	23-Aug-2020	MaT
ZMUO.070343	BOLD:AAG3236	*Diplonevra nitidula*	Turku	60.432	22.162	23-Aug-2020	MaT
ZMUO.070361	BOLD:ADM5152	*Triphleba antricola*	Turku	60.432	22.162	23-Aug-2020	MaT
ZMUO.070365	BOLD:ACB3412	*Triphleba nudipalpis*	Turku	60.432	22.162	23-Aug-2020	MaT
ZMUO.070366	BOLD:AAG3236	*Diplonevra nitidula*	Turku	60.432	22.162	23-Aug-2020	MaT
ZMUO.070443	BOLD:ACD2444	*Hypocera mordellaria*	Kuhmo	64.168	30.347	24-Jun-2020	MaT
ZMUO.070444	BOLD:ABA7001	*Diplonevra glabra*	Kuhmo	64.168	30.347	24-Jun-2020	MaT
ZMUO.070456	BOLD:AAG3249	*Phora obscura*	Kuhmo	64.168	30.347	24-Jun-2020	MaT
ZMUO.070460	BOLD:ACD2261	*Phora artifrons*	Kuhmo	64.168	30.347	24-Jun-2020	MaT
ZMUO.070477	BOLD:ACD2261	*Phora artifrons*	Kuhmo	64.168	30.347	15-Jul-2020	MaT
ZMUO.070493	BOLD:ABA7001	*Diplonevra glabra*	Kuhmo	64.168	30.347	15-Jul-2020	MaT
ZMUO.070497	BOLD:AFE7151	*Anevrina thoracica*	Kuhmo	64.168	30.347	15-Jul-2020	MaT
ZMUO.070568	BOLD:ADM5152	*Triphleba antricola*	Kuhmo	64.168	30.347	15-Aug-2020	MaT
ZMUO.070576	BOLD:ACD2464	*Borophaga carinifrons*	Kuhmo	64.168	30.347	15-Aug-2020	MaT
ZMUO.070577	BOLD:ADM5152	*Triphleba antricola*	Kuhmo	64.168	30.347	15-Aug-2020	MaT
ZMUO.070596	BOLD:AAG3236	*Diplonevra nitidula*	Kuhmo	64.168	30.347	15-Aug-2020	MaT
ZMUO.070600	BOLD:AAG3236	*Diplonevra nitidula*	Kuhmo	64.168	30.347	15-Aug-2020	MaT
ZMUO.070606	BOLD:ADM5152	*Triphleba antricola*	Kuhmo	64.168	30.347	15-Aug-2020	MaT

ZMUO.070608	BOLD:ADM5152	*Triphleba antricola*	Kuhmo	64.168	30.347	15-Aug-2020	MaT
ZMUO.070623	BOLD:ACD2464	*Borophaga carinifrons*	Kuhmo	64.168	30.347	15-Aug-2020	MaT
ZMUO.070645	BOLD:ADM5152	*Triphleba antricola*	Kuhmo	64.168	30.347	15-Aug-2020	MaT
ZMUO.070647	BOLD:AAG3236	*Diplonevra nitidula*	Kuhmo	64.168	30.347	15-Aug-2020	MaT
ZMUO.070648	BOLD:ACD2464	*Borophaga carinifrons*	Kuhmo	64.168	30.347	15-Aug-2020	MaT
ZMUO.070656	BOLD:ADM5152	*Triphleba antricola*	Kuhmo	64.168	30.347	15-Aug-2020	MaT
ZMUO.070662	BOLD:AAG3236	*Diplonevra nitidula*	Kuhmo	64.168	30.347	24-Aug-2020	MaT
ZMUO.070681	BOLD:ACD2464	*Borophaga carinifrons*	Kuhmo	64.168	30.347	24-Aug-2020	MaT
ZMUO.070690	BOLD:ADM5152	*Triphleba antricola*	Kuhmo	64.168	30.347	24-Aug-2020	MaT
ZMUO.070691	BOLD:AAG3236	*Diplonevra nitidula*	Kuhmo	64.168	30.347	24-Aug-2020	MaT
ZMUO.070697	BOLD:AAG3236	*Diplonevra nitidula*	Kuhmo	64.168	30.347	24-Aug-2020	MaT
ZMUO.070705	BOLD:ACD2464	*Borophaga carinifrons*	Kuhmo	64.168	30.347	24-Aug-2020	MaT
ZMUO.070709	BOLD:ACD2464	*Borophaga carinifrons*	Kuhmo	64.168	30.347	24-Aug-2020	MaT
ZMUO.070713	BOLD:ACD2464	*Borophaga carinifrons*	Kuhmo	64.168	30.347	24-Aug-2020	MaT
ZMUO.070717	BOLD:ADM5152	*Triphleba antricola*	Kuhmo	64.168	30.347	24-Aug-2020	MaT
ZMUO.070718	BOLD:ACD2464	*Borophaga carinifrons*	Kuhmo	64.168	30.347	24-Aug-2020	MaT
ZMUO.070736	BOLD:ACD2464	*Borophaga carinifrons*	Kuhmo	64.168	30.347	24-Aug-2020	MaT
ZMUO.070790	BOLD:ACF6067	*Phora dubia*	Kuhmo	64.166	30.309	27-Jun-2022	MaT
ZMUO.070809	BOLD:ACD2261	*Phora artifrons*	Kuhmo	64.166	30.309	27-Jun-2022	MaT
ZMUO.070818	BOLD:AFE4578	*Anevrina thoracica*	Kuhmo	64.166	30.309	27-Jun-2022	MaT
ZMUO.070826	BOLD:ACF6067	*Phora dubia*	Kuhmo	64.166	30.309	27-Jun-2022	MaT
ZMUO.070921	BOLD:ACE1464	*Phalacrotophora fasciata*	Kuhmo	64.166	30.309	29-Jul-2022	MaT
ZMUO.070982	BOLD:ACN6664	*Phora pubipes*	Kuhmo	64.166	30.309	26-Aug-2022	MaT
ZMUO.071184	BOLD:AAN8685	*Conicera dauci*	Oulu	65.137	25.885	09-Jul-2015	MaT
ZMUO.071206	BOLD:ADM5152	*Triphleba antricola*	Oulu	65.137	25.885	09-Jul-2015	MaT
ZMUO.071213	BOLD:ABA7002	*Phora tincta*	Oulu	65.137	25.885	09-Jul-2015	MaT
ZMUO.071243	BOLD:ACD2463	*Borophaga agilis*	Oulu	65.137	25.885	15-Jul-2015	MaT
ZMUO.071283	BOLD:ACD2463	*Borophaga agilis*	Oulu	65.137	25.885	15-Jul-2015	MaT
ZMUO.071290	BOLD:ACD2463	*Borophaga agilis*	Oulu	65.137	25.885	15-Jul-2015	MaT
ZMUO.071297	BOLD:ACD2463	*Borophaga agilis*	Oulu	65.137	25.885	15-Jul-2015	MaT
ZMUO.071310	BOLD:ACD2463	*Borophaga agilis*	Oulu	65.137	25.885	15-Jul-2015	MaT
ZMUO.071343	BOLD:ACD2464	*Borophaga carinifrons*	Oulu	65.137	25.885	18-Aug-2015	MaT
ZMUO.071361	BOLD:ACD2464	*Borophaga carinifrons*	Oulu	65.137	25.885	18-Aug-2015	MaT
ZMUO.071374	BOLD:ACD2463	*Borophaga agilis*	Oulu	65.137	25.885	18-Aug-2015	MaT
ZMUO.071375	BOLD:ACD2463	*Borophaga agilis*	Oulu	65.137	25.885	18-Aug-2015	MaT
ZMUO.071376	BOLD:ACD2464	*Borophaga carinifrons*	Oulu	65.137	25.885	18-Aug-2015	MaT
ZMUO.071380	BOLD:ACD2463	*Borophaga agilis*	Oulu	65.137	25.885	18-Aug-2015	MaT
ZMUO.071383	BOLD:ACD2464	*Borophaga carinifrons*	Oulu	65.137	25.885	18-Aug-2015	MaT
ZMUO.071386	BOLD:ACI8210	*Phora convergens*	Oulu	65.137	25.885	18-Aug-2015	MaT
ZMUO.071388	BOLD:ACN6664	*Phora pubipes*	Oulu	65.137	25.885	18-Aug-2015	MaT
ZMUO.071390	BOLD:ACD2463	*Borophaga agilis*	Oulu	65.137	25.885	18-Aug-2015	MaT
ZMUO.071407	BOLD:ACD2464	*Borophaga carinifrons*	Oulu	65.137	25.885	18-Aug-2015	MaT
ZMUO.071413	BOLD:ACD2464	*Borophaga carinifrons*	Oulu	65.137	25.885	18-Aug-2015	MaT
ZMUO.071463	BOLD:AAG3348	*Triphleba aequalis*	Oulu	65.137	25.851	29-Sep-2015	MaT
ZMUO.071485	BOLD:ACF0365	*Triphleba bicornuta*	Oulu	65.137	25.851	29-Sep-2015	MaT
ZMUO.071489	BOLD:AAG3348	*Triphleba aequalis*	Oulu	65.137	25.851	29-Sep-2015	MaT
ZMUO.071490	BOLD:ACF0365	*Triphleba bicornuta*	Oulu	65.137	25.851	29-Sep-2015	MaT
ZMUO.071549	BOLD:AAG3348	*Triphleba aequalis*	Oulu	65.137	25.851	30-Oct-2015	MaT

ZMUO.071583	BOLD:ACF0365	*Triphleba bicornuta*	Oulu	65.137	25.851	30-Oct-2015	MaT
ZMUO.071588	BOLD:ACF0365	*Triphleba bicornuta*	Oulu	65.137	25.851	30-Oct-2015	MaT
ZMUO.071594	BOLD:AAG3348	*Triphleba aequalis*	Oulu	65.137	25.851	30-Oct-2015	MaT
ZMUO.071637	BOLD:AFE8790	*Plectanocnema nudipes*	Oulu	65.063	25.469	25-May-2016	MaT
ZMUO.071741	BOLD:ADA0451	*Borophaga femorata*	Oulu	65.063	25.469	06-Jun-2016	MaT
ZMUO.071782	BOLD:AAP3611	*Borophaga subsultans*	Oulu	65.063	25.469	06-Jun-2016	MaT
ZMUO.071948	BOLD:ACD2464	*Borophaga carinifrons*	Oulu	65.063	25.469	08-Aug-2016	MaT
ZMUO.071964	BOLD:ACD2463	*Borophaga agilis*	Oulu	65.063	25.469	08-Aug-2016	MaT
ZMUO.071977	BOLD:ACD2463	*Borophaga agilis*	Oulu	65.063	25.469	08-Aug-2016	MaT
ZMUO.071978	BOLD:ACD2463	*Borophaga agilis*	Oulu	65.063	25.469	08-Aug-2016	MaT
ZMUO.072123	BOLD:ACE3722	*Diplonevra florescens*	Kitee	62.078	30.197	18-May-2017	MaT
ZMUO.072264	BOLD:ACD2444	*Hypocera mordellaria*	Kitee	62.078	30.197	05-Jul-2017	MaT
ZMUO.072369	BOLD:AFE7151	*Anevrina thoracica*	Kitee	62.076	30.18	23-May-2016	MaT
ZMUO.072376	BOLD:ABA7001	*Diplonevra glabra*	Kitee	62.076	30.18	23-May-2016	MaT
ZMUO.072378	BOLD:ABA7001	*Diplonevra glabra*	Kitee	62.076	30.18	23-May-2016	MaT
ZMUO.072385	BOLD:ABA7001	*Diplonevra glabra*	Kitee	62.076	30.18	23-May-2016	MaT
ZMUO.072394	BOLD:ACF6067	*Phora dubia*	Kitee	62.076	30.18	06-Jun-2016	MaT
ZMUO.072395	BOLD:ABA7001	*Diplonevra glabra*	Kitee	62.076	30.18	06-Jun-2016	MaT
ZMUO.072398	BOLD:ABA7001	*Diplonevra glabra*	Kitee	62.076	30.18	06-Jun-2016	MaT
ZMUO.072406	BOLD:ABA7001	*Diplonevra glabra*	Kitee	62.076	30.18	06-Jun-2016	MaT
ZMUO.072407	BOLD:ABA7001	*Diplonevra glabra*	Kitee	62.076	30.18	06-Jun-2016	MaT
ZMUO.072411	BOLD:ABA7001	*Diplonevra glabra*	Kitee	62.076	30.18	06-Jun-2016	MaT
ZMUO.072420	BOLD:ABA7001	*Diplonevra glabra*	Kitee	62.076	30.18	06-Jun-2016	MaT
ZMUO.072421	BOLD:ABA7001	*Diplonevra glabra*	Kitee	62.076	30.18	06-Jun-2016	MaT
ZMUO.072428	BOLD:ABA7001	*Diplonevra glabra*	Kitee	62.076	30.18	06-Jun-2016	MaT
ZMUO.072440	BOLD:ABA7001	*Diplonevra glabra*	Kitee	62.076	30.18	06-Jun-2016	MaT
ZMUO.072442	BOLD:ABA7001	*Diplonevra glabra*	Kitee	62.076	30.18	06-Jun-2016	MaT
ZMUO.072443	BOLD:ACF6067	*Phora dubia*	Kitee	62.076	30.18	06-Jun-2016	MaT
ZMUO.072455	BOLD:ABA7001	*Diplonevra glabra*	Kitee	62.076	30.18	06-Jun-2016	MaT
ZMUO.072456	BOLD:ABA7001	*Diplonevra glabra*	Kitee	62.076	30.18	06-Jun-2016	MaT
ZMUO.072479	BOLD:ACE2645	*Conicera floricola*	Kitee	62.076	30.18	21-Jul-2016	MaT
ZMUO.072557	BOLD:ACN6664	*Phora pubipes*	Kitee	62.076	30.18	01-Aug-2016	MaT
ZMUO.072662	BOLD:AAP3611	*Borophaga subsultans*	Kitee	62.088	30.223	15-Jun-2017	MaT
ZMUO.072665	BOLD:ABA7002	*Phora tincta*	Kitee	62.088	30.223	15-Jun-2017	MaT
ZMUO.072669	BOLD:ACF6067	*Phora dubia*	Kitee	62.088	30.223	24-Jun-2017	MaT
ZMUO.072694	BOLD:ABA7001	*Diplonevra glabra*	Kitee	62.088	30.223	24-Jun-2017	MaT
ZMUO.072715	BOLD:ADM5152	*Triphleba antricola*	Kitee	62.088	30.223	05-Jul-2017	MaT
ZMUO.072745	BOLD:ACE2645	*Conicera floricola*	Kitee	62.088	30.223	16-Jul-2017	MaT
ZMUO.072790	BOLD:ADM5152	*Triphleba antricola*	Kitee	62.088	30.223	24-Jul-2017	MaT
ZMUO.072826	BOLD:ADM5152	*Triphleba antricola*	Kitee	62.088	30.223	02-Aug-2017	MaT
ZMUO.073041	BOLD:ACQ9655	*Anevrina unispinosa*	Oulu	65.059	25.52	12-Jul-2022	MaT
ZMUO.073048	BOLD:AAP3611	*Borophaga subsultans*	Oulu	65.059	25.52	12-Jul-2022	MaT
ZMUO.073261	BOLD:ABA7002	*Phora tincta*	Oulu	65.059	25.52	02-Aug-2022	MaT
ZMUO.073364	BOLD:AFE7151	*Anevrina thoracica*	Oulu	65.059	25.52	02-Aug-2022	MaT
ZMUO.073398	BOLD:AEB8779	*Phora convergens*	Oulu	65.059	25.52	15-Aug-2022	MaT
ZMUO.073471	BOLD:AEH1787	*Phora occidentata*	Oulu	65.059	25.52	30-Aug-2022	MaT
ZMUO.073538	BOLD:ABA7001	*Diplonevra glabra*	Laukaa	62.223	26.099	21-Jun-2021	MaT
ZMUO.073542	BOLD:ACD2444	*Hypocera mordellaria*	Laukaa	62.223	26.099	21-Jun-2021	MaT

ZMUO.073544	BOLD:ABA7001	*Diplonevra glabra*	Laukaa	62.223	26.099	21-Jun-2021	MaT
ZMUO.073564	BOLD:ACF8062	*Triphleba papillata*	Laukaa	62.223	26.099	21-Jun-2021	MaT
ZMUO.073580	BOLD:ADM5152	*Triphleba antricola*	Laukaa	62.223	26.099	21-Jun-2021	MaT
ZMUO.073590	BOLD:ABA7001	*Diplonevra glabra*	Laukaa	62.223	26.099	21-Jun-2021	MaT
ZMUO.073592	BOLD:ABA7001	*Diplonevra glabra*	Laukaa	62.223	26.099	21-Jun-2021	MaT
ZMUO.073602	BOLD:ABA7001	*Diplonevra glabra*	Laukaa	62.223	26.099	21-Jun-2021	MaT
ZMUO.073639	BOLD:AAG3236	*Diplonevra nitidula*	Laukaa	62.223	26.099	21-Jun-2021	MaT
ZMUO.073645	BOLD:ACB3394	*Phora bullata*	Laukaa	62.223	26.099	21-Jun-2021	MaT
ZMUO.073712	BOLD:AAG3236	*Diplonevra nitidula*	Laukaa	62.223	26.099	11-Jul-2021	MaT
ZMUO.073719	BOLD:ACE1464	*Phalacrotophora fasciata*	Laukaa	62.223	26.099	11-Jul-2021	MaT
ZMUO.073723	BOLD:ADM5152	*Triphleba antricola*	Laukaa	62.223	26.099	11-Jul-2021	MaT
ZMUO.073724	BOLD:ADM5152	*Triphleba antricola*	Laukaa	62.223	26.099	11-Jul-2021	MaT
ZMUO.073736	BOLD:ACE1464	*Phalacrotophora fasciata*	Laukaa	62.223	26.099	11-Jul-2021	MaT
ZMUO.073768	BOLD:ABW2086	*Pseudacteon fennicus*	Laukaa	62.223	26.099	11-Jul-2021	MaT
ZMUO.073771	BOLD:ACE2645	*Conicera floricola*	Laukaa	62.223	26.099	11-Jul-2021	MaT
ZMUO.073792	BOLD:ACE1464	*Phalacrotophora fasciata*	Laukaa	62.223	26.099	11-Jul-2021	MaT
ZMUO.073803	BOLD:ACF4704	*Metopina oligoneura*	Laukaa	62.223	26.099	11-Jul-2021	MaT
ZMUO.073814	BOLD:ACR0978	*Pseudacteon formicarum*	Laukaa	62.223	26.099	11-Jul-2021	MaT
ZMUO.073825	BOLD:ADM5152	*Triphleba antricola*	Laukaa	62.223	26.099	11-Jul-2021	MaT
ZMUO.073836	BOLD:ACE1464	*Phalacrotophora fasciata*	Laukaa	62.223	26.099	11-Jul-2021	MaT
ZMUO.073849	BOLD:ACQ3746	*Gymnophora arcuata*	Laukaa	62.223	26.099	11-Jul-2021	MaT
ZMUO.073851	BOLD:AAG3236	*Diplonevra nitidula*	Laukaa	62.223	26.099	11-Jul-2021	MaT
ZMUO.073854	BOLD:AAG3236	*Diplonevra nitidula*	Laukaa	62.223	26.099	11-Jul-2021	MaT
ZMUO.073857	BOLD:AAG3236	*Diplonevra nitidula*	Laukaa	62.223	26.099	11-Jul-2021	MaT
ZMUO.073862	BOLD:AAG3236	*Diplonevra nitidula*	Laukaa	62.223	26.099	11-Jul-2021	MaT
ZMUO.073887	BOLD:ADM5152	*Triphleba antricola*	Laukaa	62.223	26.099	11-Jul-2021	MaT
ZMUO.073890	BOLD:ACE1464	*Phalacrotophora fasciata*	Laukaa	62.223	26.099	11-Jul-2021	MaT
ZMUO.073899	BOLD:ACF4704	*Metopina oligoneura*	Laukaa	62.223	26.099	11-Jul-2021	MaT
ZMUO.073914	BOLD:ADM5152	*Triphleba antricola*	Laukaa	62.223	26.099	11-Jul-2021	MaT
ZMUO.073923	BOLD:ADM5152	*Triphleba antricola*	Laukaa	62.223	26.099	11-Jul-2021	MaT
ZMUO.073933	BOLD:AAG3236	*Diplonevra nitidula*	Laukaa	62.223	26.099	11-Jul-2021	MaT
ZMUO.073941	BOLD:ADM5152	*Triphleba antricola*	Laukaa	62.223	26.099	11-Jul-2021	MaT
ZMUO.073944	BOLD:ACJ1099	*Conicera schnittmanni*	Laukaa	62.223	26.099	20-Jul-2021	MaT
ZMUO.073949	BOLD:ADM5152	*Triphleba antricola*	Laukaa	62.223	26.099	20-Jul-2021	MaT
ZMUO.073952	BOLD:ACD2464	*Borophaga carinifrons*	Laukaa	62.223	26.099	20-Jul-2021	MaT
ZMUO.073953	BOLD:ACF4704	*Metopina oligoneura*	Laukaa	62.223	26.099	20-Jul-2021	MaT
ZMUO.073964	BOLD:ACO9836	*Triphleba distinguenda*	Laukaa	62.223	26.099	20-Jul-2021	MaT
ZMUO.073970	BOLD:ACO7758	*Pseudacteon brevicauda*	Laukaa	62.223	26.099	20-Jul-2021	MaT
ZMUO.073976	BOLD:ADM5152	*Triphleba antricola*	Laukaa	62.223	26.099	20-Jul-2021	MaT
ZMUO.073983	BOLD:ACZ8391	*Diplonevra pilosella*	Laukaa	62.223	26.099	20-Jul-2021	MaT
ZMUO.074007	BOLD:ADM5152	*Triphleba antricola*	Laukaa	62.223	26.099	20-Jul-2021	MaT
ZMUO.074016	BOLD:ADM5152	*Triphleba antricola*	Laukaa	62.223	26.099	20-Jul-2021	MaT
ZMUO.074022	BOLD:ACD2464	*Borophaga carinifrons*	Laukaa	62.223	26.099	20-Jul-2021	MaT
ZMUO.074023	BOLD:ADM5152	*Triphleba antricola*	Laukaa	62.223	26.099	20-Jul-2021	MaT

ZMUO.074042	BOLD:ABA7001	*Diplonevra glabra*	Vartsila	62.192	30.627	21-Jun-2021	MaT
ZMUO.074050	BOLD:AAG3236	*Diplonevra nitidula*	Vartsila	62.192	30.627	21-Jun-2021	MaT
ZMUO.074056	BOLD:AAG3236	*Diplonevra nitidula*	Vartsila	62.192	30.627	21-Jun-2021	MaT
ZMUO.074060	BOLD:ABA7001	*Diplonevra glabra*	Vartsila	62.192	30.627	21-Jun-2021	MaT
ZMUO.074064	BOLD:ABA7001	*Diplonevra glabra*	Vartsila	62.192	30.627	21-Jun-2021	MaT
ZMUO.074089	BOLD:ABA7001	*Diplonevra glabra*	Vartsila	62.192	30.627	21-Jun-2021	MaT
ZMUO.074095	BOLD:ABA7001	*Diplonevra glabra*	Vartsila	62.192	30.627	21-Jun-2021	MaT
ZMUO.074096	BOLD:ABA7001	*Diplonevra glabra*	Vartsila	62.192	30.627	21-Jun-2021	MaT
ZMUO.074098	BOLD:ABA7001	*Diplonevra glabra*	Vartsila	62.192	30.627	21-Jun-2021	MaT
ZMUO.074100	BOLD:ABA7001	*Diplonevra glabra*	Vartsila	62.192	30.627	21-Jun-2021	MaT
ZMUO.074102	BOLD:AAG3236	*Diplonevra nitidula*	Vartsila	62.192	30.627	21-Jun-2021	MaT
ZMUO.074124	BOLD:AAG3236	*Diplonevra nitidula*	Vartsila	62.192	30.627	19-Jul-2021	MaT
ZMUO.074130	BOLD:ACD2463	*Borophaga agilis*	Vartsila	62.192	30.627	19-Jul-2021	MaT
ZMUO.074133	BOLD:AAG3236	*Diplonevra nitidula*	Vartsila	62.192	30.627	19-Jul-2021	MaT
ZMUO.074134	BOLD:AAG3236	*Diplonevra nitidula*	Vartsila	62.192	30.627	19-Jul-2021	MaT
ZMUO.074137	BOLD:ABA7001	*Diplonevra glabra*	Vartsila	62.192	30.627	19-Jul-2021	MaT
ZMUO.074141	BOLD:ACQ9655	*Anevrina unispinosa*	Vartsila	62.192	30.627	19-Jul-2021	MaT
ZMUO.074150	BOLD:AAG3236	*Diplonevra nitidula*	Vartsila	62.192	30.627	19-Jul-2021	MaT
ZMUO.074164	BOLD:ACB3701	*Diplonevra concinna*	Vartsila	62.192	30.627	19-Jul-2021	MaT
ZMUO.074165	BOLD:AFE7151	*Anevrina thoracica*	Vartsila	62.192	30.627	19-Jul-2021	MaT
ZMUO.074167	BOLD:AAG3236	*Diplonevra nitidula*	Vartsila	62.192	30.627	19-Jul-2021	MaT
ZMUO.074205	BOLD:ABA7001	*Diplonevra glabra*	Vartsila	62.192	30.627	19-Jul-2021	MaT
ZMUO.074208	BOLD:AAG3236	*Diplonevra nitidula*	Vartsila	62.192	30.627	19-Jul-2021	MaT
ZMUO.074209	BOLD:ACD2463	*Borophaga agilis*	Vartsila	62.192	30.627	19-Jul-2021	MaT
ZMUO.074212	BOLD:ACQ9655	*Anevrina unispinosa*	Vartsila	62.192	30.627	19-Jul-2021	MaT
ZMUO.074213	BOLD:AAG3236	*Diplonevra nitidula*	Vartsila	62.192	30.627	19-Jul-2021	MaT
ZMUO.074214	BOLD:AAG3236	*Diplonevra nitidula*	Vartsila	62.192	30.627	19-Jul-2021	MaT
ZMUO.074215	BOLD:ACQ9655	*Anevrina unispinosa*	Vartsila	62.192	30.627	19-Jul-2021	MaT
ZMUO.074217	BOLD:AAG3236	*Diplonevra nitidula*	Vartsila	62.192	30.627	19-Jul-2021	MaT
ZMUO.074228	BOLD:ABA7001	*Diplonevra glabra*	Vartsila	62.192	30.627	19-Jul-2021	MaT
ZMUO.074229	BOLD:AAG3236	*Diplonevra nitidula*	Vartsila	62.192	30.627	19-Jul-2021	MaT
ZMUO.074231	BOLD:AAG3236	*Diplonevra nitidula*	Vartsila	62.192	30.627	19-Jul-2021	MaT
ZMUO.074232	BOLD:AAG3236	*Diplonevra nitidula*	Vartsila	62.192	30.627	19-Jul-2021	MaT
ZMUO.074234	BOLD:AAG3236	*Diplonevra nitidula*	Vartsila	62.192	30.627	19-Jul-2021	MaT
ZMUO.074241	BOLD:AAG3236	*Diplonevra nitidula*	Vartsila	62.192	30.627	19-Jul-2021	MaT
ZMUO.074242	BOLD:AAG3236	*Diplonevra nitidula*	Vartsila	62.192	30.627	19-Jul-2021	MaT
ZMUO.074243	BOLD:ACD2463	*Borophaga agilis*	Vartsila	62.192	30.627	19-Jul-2021	MaT
ZMUO.074248	BOLD:AAG3236	*Diplonevra nitidula*	Vartsila	62.192	30.627	19-Jul-2021	MaT
ZMUO.074257	BOLD:AAG3236	*Diplonevra nitidula*	Vartsila	62.192	30.627	19-Jul-2021	MaT
ZMUO.074281	BOLD:AAG3236	*Diplonevra nitidula*	Vartsila	62.192	30.627	19-Jul-2021	MaT
ZMUO.074282	BOLD:AAG3236	*Diplonevra nitidula*	Vartsila	62.192	30.627	19-Jul-2021	MaT
ZMUO.074288	BOLD:AAG3236	*Diplonevra nitidula*	Vartsila	62.192	30.627	19-Jul-2021	MaT
ZMUO.074294	BOLD:AAG3236	*Diplonevra nitidula*	Vartsila	62.192	30.627	19-Jul-2021	MaT
ZMUO.074298	BOLD:ABA7001	*Diplonevra glabra*	Vartsila	62.192	30.627	19-Jul-2021	MaT
ZMUO.074309	BOLD:AAG3236	*Diplonevra nitidula*	Vartsila	62.192	30.627	19-Jul-2021	MaT
ZMUO.074311	BOLD:ACB3701	*Diplonevra concinna*	Vartsila	62.192	30.627	19-Jul-2021	MaT
ZMUO.074319	BOLD:ABA7001	*Diplonevra glabra*	Vartsila	62.192	30.627	19-Jul-2021	MaT
ZMUO.074323	BOLD:AAG3236	*Diplonevra nitidula*	Vartsila	62.192	30.627	19-Jul-2021	MaT

ZMUO.074332	BOLD:ACQ9655	*Anevrina unispinosa*	Vartsila	62.192	30.627	19-Jul-2021	MaT
ZMUO.074336	BOLD:AAG3236	*Diplonevra nitidula*	Vartsila	62.192	30.627	19-Jul-2021	MaT
ZMUO.074344	BOLD:ACD2463	*Borophaga agilis*	Vartsila	62.192	30.627	19-Jul-2021	MaT
ZMUO.074347	BOLD:ACD2463	*Borophaga agilis*	Vartsila	62.192	30.627	19-Jul-2021	MaT
ZMUO.074354	BOLD:AAG3236	*Diplonevra nitidula*	Vartsila	62.192	30.627	19-Jul-2021	MaT
ZMUO.074356	BOLD:ABA7001	*Diplonevra glabra*	Vartsila	62.192	30.627	19-Jul-2021	MaT
ZMUO.074357	BOLD:AAG3236	*Diplonevra nitidula*	Vartsila	62.192	30.627	19-Jul-2021	MaT
ZMUO.074363	BOLD:AAG3236	*Diplonevra nitidula*	Vartsila	62.192	30.627	19-Jul-2021	MaT
ZMUO.074367	BOLD:AEB8779	*Phora convergens*	Vartsila	62.192	30.627	19-Jul-2021	MaT
ZMUO.074388	BOLD:AAG3236	*Diplonevra nitidula*	Vartsila	62.192	30.627	19-Jul-2021	MaT
ZMUO.074396	BOLD:ACF4704	*Metopina oligoneura*	Parikkala	61.566	29.561	21-Jun-2021	MaT
ZMUO.074403	BOLD:ADM5152	*Triphleba antricola*	Parikkala	61.566	29.561	21-Jun-2021	MaT
ZMUO.074404	BOLD:ACB3412	*Triphleba nudipalpis*	Parikkala	61.566	29.561	21-Jun-2021	MaT
ZMUO.074410	BOLD:ACF8494	*Conicera similis*	Parikkala	61.566	29.561	21-Jun-2021	MaT
ZMUO.074414	BOLD:AAG3236	*Diplonevra nitidula*	Parikkala	61.566	29.561	21-Jun-2021	MaT
ZMUO.074419	BOLD:ACF4704	*Metopina oligoneura*	Parikkala	61.566	29.561	21-Jun-2021	MaT
ZMUO.074422	BOLD:AAG3236	*Diplonevra nitidula*	Parikkala	61.566	29.561	21-Jun-2021	MaT
ZMUO.074425	BOLD:ACF4704	*Metopina oligoneura*	Parikkala	61.566	29.561	21-Jun-2021	MaT
ZMUO.074461	BOLD:ACF4704	*Metopina oligoneura*	Parikkala	61.566	29.561	21-Jun-2021	MaT
ZMUO.074466	BOLD:ACF4704	*Metopina oligoneura*	Parikkala	61.566	29.561	21-Jun-2021	MaT
ZMUO.074473	BOLD:ACB3394	*Phora bullata*	Parikkala	61.566	29.561	21-Jun-2021	MaT
ZMUO.074485	BOLD:ACF4704	*Metopina oligoneura*	Parikkala	61.566	29.561	21-Jun-2021	MaT
ZMUO.074505	BOLD:ACE2645	*Conicera floricola*	Parikkala	61.566	29.561	21-Jun-2021	MaT
ZMUO.074507	BOLD:ACF8494	*Conicera similis*	Parikkala	61.566	29.561	21-Jun-2021	MaT
ZMUO.074516	BOLD:ACF4704	*Metopina oligoneura*	Parikkala	61.566	29.561	21-Jun-2021	MaT
ZMUO.074518	BOLD:ADM5152	*Triphleba antricola*	Parikkala	61.566	29.561	21-Jun-2021	MaT
ZMUO.074535	BOLD:ACF4704	*Metopina oligoneura*	Parikkala	61.566	29.561	21-Jun-2021	MaT
ZMUO.074537	BOLD:ACR0978	*Pseudacteon formicarum*	Parikkala	61.566	29.561	21-Jun-2021	MaT
ZMUO.074545	BOLD:ACF4704	*Metopina oligoneura*	Parikkala	61.566	29.561	21-Jun-2021	MaT
ZMUO.074547	BOLD:ACE2645	*Conicera floricola*	Parikkala	61.566	29.561	21-Jun-2021	MaT
ZMUO.074549	BOLD:ACF4704	*Metopina oligoneura*	Parikkala	61.566	29.561	21-Jun-2021	MaT
ZMUO.074561	BOLD:ACF4704	*Metopina oligoneura*	Parikkala	61.566	29.561	21-Jun-2021	MaT
ZMUO.074562	BOLD:ACF4704	*Metopina oligoneura*	Parikkala	61.566	29.561	21-Jun-2021	MaT
ZMUO.074563	BOLD:ACF4704	*Metopina oligoneura*	Parikkala	61.566	29.561	21-Jun-2021	MaT
ZMUO.074567	BOLD:ACF4704	*Metopina oligoneura*	Parikkala	61.566	29.561	21-Jun-2021	MaT
ZMUO.074569	BOLD:ACF4704	*Metopina oligoneura*	Parikkala	61.566	29.561	21-Jun-2021	MaT
ZMUO.074571	BOLD:ACR0978	*Pseudacteon formicarum*	Parikkala	61.566	29.561	21-Jun-2021	MaT
ZMUO.074572	BOLD:ACF4704	*Metopina oligoneura*	Parikkala	61.566	29.561	21-Jun-2021	MaT
ZMUO.074576	BOLD:ACF4704	*Metopina oligoneura*	Parikkala	61.566	29.561	21-Jun-2021	MaT
ZMUO.074581	BOLD:ACF4704	*Metopina oligoneura*	Parikkala	61.566	29.561	21-Jun-2021	MaT
ZMUO.074586	BOLD:ACF4704	*Metopina oligoneura*	Parikkala	61.566	29.561	21-Jun-2021	MaT
ZMUO.074590	BOLD:ACF4704	*Metopina oligoneura*	Parikkala	61.566	29.561	21-Jun-2021	MaT
ZMUO.074595	BOLD:ACF4704	*Metopina oligoneura*	Parikkala	61.566	29.561	21-Jun-2021	MaT
ZMUO.074598	BOLD:AAG3236	*Diplonevra nitidula*	Parikkala	61.566	29.561	21-Jun-2021	MaT
ZMUO.074599	BOLD:ACF4704	*Metopina oligoneura*	Parikkala	61.566	29.561	21-Jun-2021	MaT
ZMUO.074608	BOLD:ACF4704	*Metopina oligoneura*	Parikkala	61.566	29.561	21-Jun-2021	MaT
ZMUO.074612	BOLD:AAG3236	*Diplonevra nitidula*	Parikkala	61.566	29.561	21-Jun-2021	MaT

ZMUO.074621	BOLD:ACF4704	*Metopina oligoneura*	Parikkala	61.566	29.561	21-Jun-2021	MaT
ZMUO.074624	BOLD:ACF4704	*Metopina oligoneura*	Parikkala	61.566	29.561	21-Jun-2021	MaT
ZMUO.074638	BOLD:ACF4704	*Metopina oligoneura*	Parikkala	61.566	29.561	21-Jun-2021	MaT
ZMUO.074639	BOLD:ACF4704	*Metopina oligoneura*	Parikkala	61.566	29.561	21-Jun-2021	MaT
ZMUO.074640	BOLD:ACF4704	*Metopina oligoneura*	Parikkala	61.566	29.561	21-Jun-2021	MaT
ZMUO.074648	BOLD:ACF4704	*Metopina oligoneura*	Parikkala	61.566	29.561	21-Jun-2021	MaT
ZMUO.074657	BOLD:ADM5152	*Triphleba antricola*	Parikkala	61.566	29.561	21-Jun-2021	MaT
ZMUO.074661	BOLD:ACF4704	*Metopina oligoneura*	Parikkala	61.566	29.561	21-Jun-2021	MaT
ZMUO.074666	BOLD:ACP3737	*Phora edentata*	Parikkala	61.566	29.561	21-Jun-2021	MaT
ZMUO.074674	BOLD:ACF4704	*Metopina oligoneura*	Parikkala	61.566	29.561	21-Jun-2021	MaT
ZMUO.074677	BOLD:ACF4704	*Metopina oligoneura*	Parikkala	61.566	29.561	21-Jun-2021	MaT
ZMUO.074680	BOLD:ACF4704	*Metopina oligoneura*	Parikkala	61.566	29.561	21-Jun-2021	MaT
ZMUO.074681	BOLD:ACF4704	*Metopina oligoneura*	Parikkala	61.566	29.561	21-Jun-2021	MaT
ZMUO.074682	BOLD:ACF4704	*Metopina oligoneura*	Parikkala	61.566	29.561	21-Jun-2021	MaT
ZMUO.074687	BOLD:ACF4704	*Metopina oligoneura*	Parikkala	61.566	29.561	21-Jun-2021	MaT
ZMUO.074702	BOLD:ACF4704	*Metopina oligoneura*	Parikkala	61.566	29.561	21-Jun-2021	MaT
ZMUO.074704	BOLD:ACF4704	*Metopina oligoneura*	Parikkala	61.566	29.561	21-Jun-2021	MaT
ZMUO.074708	BOLD:ACF4704	*Metopina oligoneura*	Parikkala	61.566	29.561	21-Jun-2021	MaT
ZMUO.074714	BOLD:ADW4894	*Pseudacteon lundbecki*	Parikkala	61.566	29.561	21-Jun-2021	MaT
ZMUO.074718	BOLD:ACF4704	*Metopina oligoneura*	Parikkala	61.566	29.561	21-Jun-2021	MaT
ZMUO.074720	BOLD:ACF4704	*Metopina oligoneura*	Parikkala	61.566	29.561	21-Jun-2021	MaT
ZMUO.074721	BOLD:ABA7002	*Phora tincta*	Parikkala	61.566	29.561	21-Jun-2021	MaT
ZMUO.074724	BOLD:ACF4704	*Metopina oligoneura*	Parikkala	61.566	29.561	21-Jun-2021	MaT
ZMUO.074727	BOLD:ACF4704	*Metopina oligoneura*	Parikkala	61.566	29.561	21-Jun-2021	MaT
ZMUO.074729	BOLD:ACF4704	*Metopina oligoneura*	Parikkala	61.566	29.561	21-Jun-2021	MaT
ZMUO.074731	BOLD:ACF4704	*Metopina oligoneura*	Parikkala	61.566	29.561	21-Jun-2021	MaT
ZMUO.074733	BOLD:ACD2463	*Borophaga agilis*	Parikkala	61.566	29.561	21-Jun-2021	MaT
ZMUO.074740	BOLD:ACF4704	*Metopina oligoneura*	Parikkala	61.566	29.561	21-Jun-2021	MaT
ZMUO.074743	BOLD:ACF4704	*Metopina oligoneura*	Parikkala	61.566	29.561	21-Jun-2021	MaT
ZMUO.074746	BOLD:ACF4704	*Metopina oligoneura*	Parikkala	61.566	29.561	21-Jun-2021	MaT
ZMUO.074751	BOLD:ADM5152	*Triphleba antricola*	Parikkala	61.566	29.561	21-Jun-2021	MaT
ZMUO.074754	BOLD:ACF4704	*Metopina oligoneura*	Parikkala	61.566	29.561	21-Jun-2021	MaT
ZMUO.074755	BOLD:ACF4704	*Metopina oligoneura*	Parikkala	61.566	29.561	21-Jun-2021	MaT
ZMUO.074757	BOLD:ACF4704	*Metopina oligoneura*	Parikkala	61.566	29.561	21-Jun-2021	MaT
ZMUO.074766	BOLD:ACF4704	*Metopina oligoneura*	Parikkala	61.566	29.561	21-Jun-2021	MaT
ZMUO.074768	BOLD:ACF4704	*Metopina oligoneura*	Parikkala	61.566	29.561	21-Jun-2021	MaT
ZMUO.074769	BOLD:ACF4704	*Metopina oligoneura*	Parikkala	61.566	29.561	21-Jun-2021	MaT
ZMUO.074773	BOLD:ACF4704	*Metopina oligoneura*	Parikkala	61.566	29.561	21-Jun-2021	MaT
ZMUO.074777	BOLD:ACF4704	*Metopina oligoneura*	Parikkala	61.566	29.561	21-Jun-2021	MaT
ZMUO.074778	BOLD:ACF4704	*Metopina oligoneura*	Parikkala	61.566	29.561	21-Jun-2021	MaT
ZMUO.074781	BOLD:ACF4704	*Metopina oligoneura*	Parikkala	61.566	29.561	21-Jun-2021	MaT
ZMUO.074782	BOLD:ACB3871	*Aenigmatias lubbockii*	Parikkala	61.566	29.561	21-Jun-2021	MaT
ZMUO.074785	BOLD:ACF4704	*Metopina oligoneura*	Parikkala	61.566	29.561	21-Jun-2021	MaT
ZMUO.074789	BOLD:AAG3236	*Diplonevra nitidula*	Parikkala	61.566	29.561	21-Jun-2021	MaT
ZMUO.074796	BOLD:ACB3701	*Diplonevra concinna*	Parikkala	61.566	29.561	21-Jun-2021	MaT
ZMUO.074797	BOLD:ACP3737	*Phora edentata*	Parikkala	61.566	29.561	21-Jun-2021	MaT
ZMUO.074808	BOLD:ACF4704	*Metopina oligoneura*	Parikkala	61.566	29.561	19-Jul-2021	MaT
ZMUO.074819	BOLD:ACF4704	*Metopina oligoneura*	Parikkala	61.566	29.561	19-Jul-2021	MaT

ZMUO.074831	BOLD:ACF4704	*Metopina oligoneura*	Parikkala	61.566	29.561	19-Jul-2021	MaT
ZMUO.074834	BOLD:ADM8144	*Phora obscura*	Parikkala	61.566	29.561	19-Jul-2021	MaT
ZMUO.074848	BOLD:ACP3737	*Phora edentata*	Parikkala	61.566	29.561	19-Jul-2021	MaT
ZMUO.074862	BOLD:ACD5037	*Triphleba nudipalpis*	Parikkala	61.566	29.561	19-Jul-2021	MaT
ZMUO.074864	BOLD:ADM5152	*Triphleba antricola*	Parikkala	61.566	29.561	19-Jul-2021	MaT
ZMUO.074867	BOLD:ACD2261	*Phora artifrons*	Parikkala	61.566	29.561	19-Jul-2021	MaT
ZMUO.074879	BOLD:ADM5152	*Triphleba antricola*	Parikkala	61.566	29.561	19-Jul-2021	MaT
ZMUO.074881	BOLD:ABA7001	*Diplonevra glabra*	Parikkala	61.566	29.561	19-Jul-2021	MaT
ZMUO.074885	BOLD:ACB3394	*Phora bullata*	Parikkala	61.566	29.561	19-Jul-2021	MaT
ZMUO.074896	BOLD:ACF4704	*Metopina oligoneura*	Parikkala	61.566	29.561	19-Jul-2021	MaT
ZMUO.074898	BOLD:ACB3412	*Triphleba nudipalpis*	Parikkala	61.566	29.561	19-Jul-2021	MaT
ZMUO.074902	BOLD:ACF4704	*Metopina oligoneura*	Parikkala	61.566	29.561	19-Jul-2021	MaT
ZMUO.074904	BOLD:ACF4704	*Metopina oligoneura*	Parikkala	61.566	29.561	19-Jul-2021	MaT
ZMUO.074907	BOLD:ACF6067	*Phora dubia*	Parikkala	61.566	29.561	19-Jul-2021	MaT
ZMUO.074912	BOLD:ACF4704	*Metopina oligoneura*	Parikkala	61.566	29.561	19-Jul-2021	MaT
ZMUO.074914	BOLD:ACF6067	*Phora dubia*	Parikkala	61.566	29.561	19-Jul-2021	MaT
ZMUO.074918	BOLD:ADM5152	*Triphleba antricola*	Parikkala	61.566	29.561	19-Jul-2021	MaT
ZMUO.074929	BOLD:ACF4704	*Metopina oligoneura*	Parikkala	61.566	29.561	19-Jul-2021	MaT
ZMUO.074949	BOLD:ADM5152	*Triphleba antricola*	Parikkala	61.566	29.561	19-Jul-2021	MaT
ZMUO.074974	BOLD:ACF4704	*Metopina oligoneura*	Parikkala	61.566	29.561	19-Jul-2021	MaT
ZMUO.074982	BOLD:ACF4704	*Metopina oligoneura*	Parikkala	61.566	29.561	19-Jul-2021	MaT
ZMUO.074987	BOLD:ACF6067	*Phora dubia*	Parikkala	61.566	29.561	19-Jul-2021	MaT
ZMUO.074989	BOLD:ABA7002	*Phora tincta*	Parikkala	61.566	29.561	19-Jul-2021	MaT
ZMUO.074990	BOLD:ACF6067	*Phora dubia*	Parikkala	61.566	29.561	19-Jul-2021	MaT
ZMUO.074992	BOLD:ACB3412	*Triphleba nudipalpis*	Parikkala	61.566	29.561	19-Jul-2021	MaT
ZMUO.074996	BOLD:ACB3394	*Phora bullata*	Parikkala	61.566	29.561	19-Jul-2021	MaT
ZMUO.075025	BOLD:ACB3412	*Triphleba nudipalpis*	Parikkala	61.566	29.561	19-Jul-2021	MaT
ZMUO.075026	BOLD:ACF6067	*Phora dubia*	Parikkala	61.566	29.561	19-Jul-2021	MaT
ZMUO.075047	BOLD:ACF4704	*Metopina oligoneura*	Parikkala	61.566	29.561	19-Jul-2021	MaT
ZMUO.075053	BOLD:ADW4894	*Pseudacteon lundbecki*	Parikkala	61.566	29.561	19-Jul-2021	MaT
ZMUO.075059	BOLD:ACB3412	*Triphleba nudipalpis*	Parikkala	61.566	29.561	19-Jul-2021	MaT
ZMUO.075067	BOLD:ACF6067	*Phora dubia*	Parikkala	61.566	29.561	19-Jul-2021	MaT
ZMUO.075072	BOLD:ACE2645	*Conicera floricola*	Parikkala	61.566	29.561	19-Jul-2021	MaT
ZMUO.075074	BOLD:ACP3737	*Phora edentata*	Parikkala	61.566	29.561	19-Jul-2021	MaT
ZMUO.075081	BOLD:ADM8636	*Phora hamata*	Parikkala	61.566	29.561	19-Jul-2021	MaT
ZMUO.075099	BOLD:ACF6067	*Phora dubia*	Parikkala	61.566	29.561	19-Jul-2021	MaT
ZMUO.075115	BOLD:ABA7002	*Phora tincta*	Parikkala	61.566	29.561	19-Jul-2021	MaT
ZMUO.075123	BOLD:ACE2645	*Conicera floricola*	Parikkala	61.566	29.561	19-Jul-2021	MaT
ZMUO.075145	BOLD:ADM5152	*Triphleba antricola*	Parikkala	61.566	29.561	19-Jul-2021	MaT
ZMUO.075153	BOLD:ACF4704	*Metopina oligoneura*	Parikkala	61.566	29.561	19-Jul-2021	MaT
ZMUO.075155	BOLD:ACF4704	*Metopina oligoneura*	Parikkala	61.566	29.561	19-Jul-2021	MaT
ZMUO.075156	BOLD:ACB3412	*Triphleba nudipalpis*	Parikkala	61.566	29.561	19-Jul-2021	MaT
ZMUO.075177	BOLD:ACF6067	*Phora dubia*	Parikkala	61.566	29.561	19-Jul-2021	MaT
ZMUO.075183	BOLD:ABA7002	*Phora tincta*	Parikkala	61.566	29.561	19-Jul-2021	MaT
ZMUO.075190	BOLD:ADM5152	*Triphleba antricola*	Parikkala	61.566	29.561	19-Jul-2021	MaT
ZMUO.075191	BOLD:ADM5152	*Triphleba antricola*	Parikkala	61.566	29.561	19-Jul-2021	MaT
ZMUO.075208	BOLD:ABA7001	*Diplonevra glabra*	Parikkala	61.566	29.561	19-Jul-2021	MaT
ZMUO.075226	BOLD:ACF6067	*Phora dubia*	Parikkala	61.566	29.561	19-Jul-2021	MaT

ZMUO.075238	BOLD:ADM5152	*Triphleba antricola*	Parikkala	61.566	29.561	19-Jul-2021	MaT
ZMUO.075272	BOLD:ACD2261	*Phora artifrons*	Saarijarvi	62.842	25.483	22-Jun-2021	MaT
ZMUO.075554	BOLD:ACR0978	*Pseudacteon formicarum*	Kitee	62.045	30.261	24-Jul-2016	MaT
ZMUO.075604	BOLD:ACR0791	*Triphleba lugubris*	Kitee	62.045	30.261	30-Jul-2016	MaT
ZMUO.075873	BOLD:ACB3653	*Diplonevra freyi*	Kitee	62.045	30.261	19-Jul-2016	MaT
ZMUO.075970	BOLD:ACB3394	*Phora bullata*	Kitee	62.045	30.261	19-Jul-2016	MaT
ZMUO.076023	BOLD:ACO9836	*Triphleba distinguenda*	Kitee	62.045	30.261	19-Jul-2016	MaT
ZMUO.076032	BOLD:ACO7758	*Pseudacteon brevicauda*	Kitee	62.045	30.261	19-Jul-2016	MaT
ZMUO.076046	BOLD:ACD2464	*Borophaga carinifrons*	Kitee	62.045	30.261	19-Jul-2016	MaT
ZMUO.076088	BOLD:ACI8210	*Phora convergens*	Kitee	62.045	30.261	22-Aug-2016	MaT
ZMUO.076246	BOLD:ABA7001	*Diplonevra glabra*	Kitee	62.045	30.261	25-May-2016	MaT
ZMUO.076258	BOLD:ACF6067	*Phora dubia*	Kitee	62.045	30.261	25-May-2016	MaT
ZMUO.076286	BOLD:ADM5152	*Triphleba antricola*	Lappeenranta	61.061	28.745	21-Jun-2021	MaT
ZMUO.076289	BOLD:ABA7001	*Diplonevra glabra*	Lappeenranta	61.061	28.745	21-Jun-2021	MaT
ZMUO.076292	BOLD:ABA7001	*Diplonevra glabra*	Lappeenranta	61.061	28.745	21-Jun-2021	MaT
ZMUO.076304	BOLD:ACF6067	*Phora dubia*	Lappeenranta	61.061	28.745	21-Jun-2021	MaT
ZMUO.076313	BOLD:ACF6067	*Phora dubia*	Lappeenranta	61.061	28.745	21-Jun-2021	MaT
ZMUO.076328	BOLD:ABA7001	*Diplonevra glabra*	Lappeenranta	61.061	28.745	21-Jun-2021	MaT
ZMUO.076329	BOLD:ADM5152	*Triphleba antricola*	Lappeenranta	61.061	28.745	21-Jun-2021	MaT
ZMUO.076335	BOLD:AAG3236	*Diplonevra nitidula*	Lappeenranta	61.061	28.745	21-Jun-2021	MaT
ZMUO.076336	BOLD:ACD2261	*Phora artifrons*	Lappeenranta	61.061	28.745	21-Jun-2021	MaT
ZMUO.076337	BOLD:ABA7002	*Phora tincta*	Lappeenranta	61.061	28.745	21-Jun-2021	MaT
ZMUO.076338	BOLD:ACD2261	*Phora artifrons*	Lappeenranta	61.061	28.745	21-Jun-2021	MaT
ZMUO.076348	BOLD:ACD2261	*Phora artifrons*	Lappeenranta	61.061	28.745	21-Jun-2021	MaT
ZMUO.076354	BOLD:ACD2261	*Phora artifrons*	Lappeenranta	61.061	28.745	21-Jun-2021	MaT
ZMUO.076355	BOLD:ADM5152	*Triphleba antricola*	Lappeenranta	61.061	28.745	21-Jun-2021	MaT
ZMUO.076356	BOLD:ABA7002	*Phora tincta*	Lappeenranta	61.061	28.745	21-Jun-2021	MaT
ZMUO.076358	BOLD:ACD2261	*Phora artifrons*	Lappeenranta	61.061	28.745	21-Jun-2021	MaT
ZMUO.076368	BOLD:ACD2261	*Phora artifrons*	Lappeenranta	61.061	28.745	21-Jun-2021	MaT
ZMUO.076372	BOLD:ADM5152	*Triphleba antricola*	Lappeenranta	61.061	28.745	21-Jun-2021	MaT
ZMUO.076373	BOLD:ADM5152	*Triphleba antricola*	Lappeenranta	61.061	28.745	21-Jun-2021	MaT
ZMUO.076391	BOLD:ADM5152	*Triphleba antricola*	Lappeenranta	61.061	28.745	21-Jun-2021	MaT
ZMUO.076417	BOLD:ACD2261	*Phora artifrons*	Lappeenranta	61.061	28.745	21-Jun-2021	MaT
ZMUO.076419	BOLD:ABA7001	*Diplonevra glabra*	Lappeenranta	61.061	28.745	21-Jun-2021	MaT
ZMUO.076422	BOLD:ABA7001	*Diplonevra glabra*	Lappeenranta	61.061	28.745	21-Jun-2021	MaT
ZMUO.076454	BOLD:AAG3236	*Diplonevra nitidula*	Lappeenranta	61.061	28.745	21-Jun-2021	MaT
ZMUO.076463	BOLD:ADM5152	*Triphleba antricola*	Lappeenranta	61.061	28.745	21-Jun-2021	MaT
ZMUO.076470	BOLD:ACD2261	*Phora artifrons*	Lappeenranta	61.061	28.745	21-Jun-2021	MaT
ZMUO.076474	BOLD:ADM5152	*Triphleba antricola*	Lappeenranta	61.061	28.745	21-Jun-2021	MaT
ZMUO.076488	BOLD:ADM5152	*Triphleba antricola*	Lappeenranta	61.061	28.745	21-Jun-2021	MaT
ZMUO.076499	BOLD:ABA7002	*Phora tincta*	Lappeenranta	61.061	28.745	19-Jul-2021	MaT
ZMUO.076513	BOLD:ABA7001	*Diplonevra glabra*	Lappeenranta	61.061	28.745	19-Jul-2021	MaT
ZMUO.076516	BOLD:AAP9864	*Triphleba subcompleta*	Lappeenranta	61.061	28.745	19-Jul-2021	MaT
ZMUO.076525	BOLD:AAG3236	*Diplonevra nitidula*	Lappeenranta	61.061	28.745	19-Jul-2021	MaT
ZMUO.076530	BOLD:ADM5152	*Triphleba antricola*	Lappeenranta	61.061	28.745	19-Jul-2021	MaT
ZMUO.076541	BOLD:AAG3236	*Diplonevra nitidula*	Lappeenranta	61.061	28.745	19-Jul-2021	MaT
ZMUO.076544	BOLD:AAG3236	*Diplonevra nitidula*	Lappeenranta	61.061	28.745	19-Jul-2021	MaT

ZMUO.076552	BOLD:ADM5152	*Triphleba antricola*	Lappeenranta	61.061	28.745	19-Jul-2021	MaT
ZMUO.076554	BOLD:ACZ8391	*Diplonevra pilosella*	Lappeenranta	61.061	28.745	19-Jul-2021	MaT
ZMUO.076561	BOLD:ACD2463	*Borophaga agilis*	Lappeenranta	61.061	28.745	19-Jul-2021	MaT
ZMUO.076564	BOLD:AAP9864	*Triphleba subcompleta*	Lappeenranta	61.061	28.745	19-Jul-2021	MaT
ZMUO.076568	BOLD:AAG3236	*Diplonevra nitidula*	Lappeenranta	61.061	28.745	19-Jul-2021	MaT
ZMUO.076569	BOLD:ADM5152	*Triphleba antricola*	Lappeenranta	61.061	28.745	19-Jul-2021	MaT
ZMUO.076586	BOLD:AAG3236	*Diplonevra nitidula*	Lappeenranta	61.061	28.745	19-Jul-2021	MaT
ZMUO.076597	BOLD:ADM5152	*Triphleba antricola*	Lappeenranta	61.061	28.745	19-Jul-2021	MaT
ZMUO.076599	BOLD:ABA7002	*Phora tincta*	Lappeenranta	61.061	28.745	19-Jul-2021	MaT
ZMUO.076609	BOLD:ABA7002	*Phora tincta*	Lappeenranta	61.061	28.745	19-Jul-2021	MaT
ZMUO.076617	BOLD:AAG3236	*Diplonevra nitidula*	Lappeenranta	61.061	28.745	19-Jul-2021	MaT
ZMUO.076631	BOLD:AAG3236	*Diplonevra nitidula*	Lappeenranta	61.061	28.745	19-Jul-2021	MaT
ZMUO.076633	BOLD:ADM5152	*Triphleba antricola*	Lappeenranta	61.061	28.745	19-Jul-2021	MaT
ZMUO.076635	BOLD:ACD2444	*Hypocera mordellaria*	Lappeenranta	61.061	28.745	19-Jul-2021	MaT
ZMUO.076641	BOLD:ACD2463	*Borophaga agilis*	Lappeenranta	61.061	28.745	19-Jul-2021	MaT
ZMUO.076666	BOLD:ADM5152	*Triphleba antricola*	Lappeenranta	61.061	28.745	19-Jul-2021	MaT
ZMUO.076682	BOLD:ADM5152	*Triphleba antricola*	Lappeenranta	61.061	28.745	19-Jul-2021	MaT
ZMUO.076685	BOLD:ACI8210	*Phora convergens*	Lappeenranta	61.061	28.745	19-Jul-2021	MaT
ZMUO.076703	BOLD:ACN6664	*Phora pubipes*	Lappeenranta	61.061	28.745	19-Jul-2021	MaT
ZMUO.076711	BOLD:ACQ9655	*Anevrina unispinosa*	Lappeenranta	61.061	28.745	19-Jul-2021	MaT
ZMUO.076724	BOLD:ACN6664	*Phora pubipes*	Lappeenranta	61.061	28.745	19-Jul-2021	MaT
ZMUO.076729	BOLD:ADM5152	*Triphleba antricola*	Lappeenranta	61.061	28.745	19-Jul-2021	MaT
ZMUO.076730	BOLD:ACN6664	*Phora pubipes*	Lappeenranta	61.061	28.745	19-Jul-2021	MaT
ZMUO.076745	BOLD:AAP9864	*Triphleba subcompleta*	Lappeenranta	61.061	28.745	19-Jul-2021	MaT
ZMUO.076753	BOLD:ADM5152	*Triphleba antricola*	Lappeenranta	61.061	28.745	19-Jul-2021	MaT
ZMUO.076756	BOLD:ADM5152	*Triphleba antricola*	Lappeenranta	61.061	28.745	19-Jul-2021	MaT
ZMUO.076760	BOLD:ADM5152	*Triphleba antricola*	Lappeenranta	61.061	28.745	19-Jul-2021	MaT
ZMUO.076763	BOLD:ACD2463	*Borophaga agilis*	Lappeenranta	61.061	28.745	19-Jul-2021	MaT
ZMUO.076813	BOLD:AFE7151	*Anevrina thoracica*	Lappeenranta	61.063	28.735	21-Jun-2021	MaT
ZMUO.076837	BOLD:ACD2261	*Phora artifrons*	Lappeenranta	61.063	28.735	21-Jun-2021	MaT
ZMUO.076860	BOLD:ACQ9655	*Anevrina unispinosa*	Lappeenranta	61.063	28.735	21-Jun-2021	MaT
ZMUO.076892	BOLD:ACF6067	*Phora dubia*	Lappeenranta	61.063	28.735	21-Jun-2021	MaT
ZMUO.076910	BOLD:ABA7002	*Phora tincta*	Lappeenranta	61.063	28.735	21-Jun-2021	MaT
ZMUO.076914	BOLD:ACF6067	*Phora dubia*	Lappeenranta	61.063	28.735	21-Jun-2021	MaT
ZMUO.076958	BOLD:ACE2645	*Conicera floricola*	Lappeenranta	61.063	28.735	19-Jul-2021	MaT
ZMUO.076963	BOLD:ACR0978	*Pseudacteon formicarum*	Lappeenranta	61.063	28.735	19-Jul-2021	MaT
ZMUO.076996	BOLD:AFE7151	*Anevrina thoracica*	Lappeenranta	61.063	28.735	19-Jul-2021	MaT
ZMUO.077036	BOLD:AAP9864	*Triphleba subcompleta*	Lappeenranta	61.063	28.735	19-Jul-2021	MaT
ZMUO.077099	BOLD:ACB3701	*Diplonevra concinna*	Lappeenranta	61.063	28.735	19-Jul-2021	MaT
