# Supplementary material for: Megabarcoding dark taxa – Assessing the utility of mass DNA barcoding for phorid fly species discovery
Source: PLoS One. 2025 Dec 2;20(12):e0334948. doi: 10.1371/journal.pone.0334948 (PMC12671794; doi:10.1371/journal.pone.0334948)
Supplement: S2 Table — Species identified using BOLD Identification Engine with >98% match, with the number of specimens from which COI barcode was recovered. Retrieved November 2023. (PDF) [file pone.0334948.s002.pdf]

S-Table 2. Initial BOLD Identification Engine results. Species identified using BOLD Identification Engine with >98% match, with the number of specimens from which *COI* barcode was recovered. Retrieved November 2023.

Species	Specimen count
Aenigmatias lubbockii	1
Anevrina thoracica	7
Borophaga agilis	21
Borophaga carinifrons	50
Borophaga femorata	1
Borophaga subsultans	3
Chaetopleurophora erythronota	1
Conicera dauci	1
Conicera floricola	9
Conicera schnittmanni	1
Conicera similis	2
Diplonevra concinna	12
Diplonevra florescens	1
Diplonevra freyi	1
Diplonevra glabra	52
Diplonevra nitidula	66
Gymnophora arcuata	1
Hypocera mordellaria	4
Metopina oligoneura	76
Phalacrotophora fasciata	6
Phora artifrons	27
Phora atra	1
Phora convergens	37
Phora dubia	10
Phora edentata	4
Phora hamata	1
Phora holosericea	5
Phora obscura	2
Phora occidentata	1
Phora pubipes	131
Phora stictica	1
Phora tincta	108
Pseudacteon brevicauda	2
Pseudacteon fennicus	1
Spiniphora excisa	2
Triphleba aequalis	4
Triphleba bicornuta	4
Triphleba citreiformis	1
Triphleba distinguenda	30
Triphleba lugubris	93
Triphleba nudipalpis	9
Triphleba papillata	1
Triphleba subcompleta	7
