# Supplementary material for: Megabarcoding dark taxa – Assessing the utility of mass DNA barcoding for phorid fly species discovery
Source: PLoS One. 2025 Dec 2;20(12):e0334948. doi: 10.1371/journal.pone.0334948 (PMC12671794; doi:10.1371/journal.pone.0334948)
Supplement: S3 Table — (PDF) [file pone.0334948.s003.pdf]

S-Table 3. Known species representation among samples.

The Finnish Checklist	Malaise trap material
Abaristophora arctophila	Not found
Abaristophora kolaensis	Not found
Aenigmatias franzi	Not found
Aenigmatias lubbockii	Present
Aenigmatias picipes	Not found
Anevrina thoracica	Present
Anevrina unispinosa	Present
Anevrina urbana	Not found
Borophaga agilis	Present
Borophaga bennetti	Not found
Borophaga carinifrons	Present
Borophaga femorata	Present
Borophaga incrassata	Not found
Borophaga irregularis	Not found
Borophaga subsultans	Present
Chaetopleurophora bohemanni	Not found
Chaetopleurophora erythronota	Present
Chaetopleurophora spinosissima	Not found
Conicera dauci	Present
Conicera floricola	Present
Conicera schnittmanni	Present
Conicera similis	Present
Conicera tarsalis	Not found
Conicera tibialis	Not found
Diplonevra concinna	Present
Diplonevra florescens	Present
Diplonevra freyi	Present
Diplonevra funebris	Not found
Diplonevra glabra	Present
Diplonevra nitidula	Present
Diplonevra oldenbergi	Not found
Diplonevra pilosella	Present
Dohrniphora cornuta	Not found
Gymnophora arcuata	Present
Gymnophora bifida	Not found
Gymnophora distincta	Not found
Gymnophora forresteri	Not found
Gymnophora healeyae	Not found
Gymnophora nigripennis	Not found
Gymnophora perpropinqua	Not found
Gymnophora quartomollis	Not found
Gymnophora winqvisti	Not found

Gymnoptera longicostalis	Not found
Hypocera mordellaria	Present
Menozziola obscuripes	Not found
Menozziola schmitzi	Not found
Metopina galeata	Not found
Metopina oligoneura	Present
Metopina pileata	Not found
Microselia forsiusi	Not found
Phalacrotophora berolinensis	Not found
Phalacrotophora beuki	Not found
Phalacrotophora fasciata	Present
Phora artifrons	Present
Phora atra	Present
Phora bullata	Present
Phora convallium	Not found
Phora convergens	Present
Phora dubia	Present
Phora edentata	Present
Phora hamata	Present
Phora holosericea	Present
Phora hyperborea	Not found
Phora indivisa	Not found
Phora obscura	Present
Phora occidentata	Present
Phora penicillata	Not found
Phora praepandens	Not found
Phora pubipes	Present
Phora stictica	Present
Phora tincta	Present
Plectanocnema nudipes	Present
Pseudacteon fennicus	Present
Pseudacteon formicarum	Present
Pseudacteon lundbecki	Present
Spiniphora bergenstammi	Not found
Spiniphora dorsalis	Not found
Spiniphora excisa	Present
Spiniphora jugorum	Not found
Spiniphora maculata	Not found
Triphleba admirabilis	Not found
Triphleba aequalis	Present
Triphleba antricola	Present
Triphleba autumnalis	Not found
Triphleba bicornuta	Present
Triphleba citreiformis	Present

Triphleba cumsetae	Not found
Triphleba dentata	Not found
Triphleba distinguenda	Present
Triphleba excisa	Not found
Triphleba gilvipes	Not found
Triphleba gracilis	Not found
Triphleba hyalinata	Not found
Triphleba inaequalis	Not found
Triphleba intermedia	Not found
Triphleba lugubris	Present
Triphleba luteifemorata	Not found
Triphleba minuta	Not found
Triphleba nudipalpis	Present
Triphleba opaca	Not found
Triphleba pachyneurella	Not found
Triphleba palposa	Not found
Triphleba papillata	Present
Triphleba renidens	Not found
Triphleba salmelai	Not found
Triphleba subcompleta	Present
Triphleba sunnmorkensis	Not found
Triphleba trinervis	Not found
Trucidophora ewardurskae	Not found
Veruanus oldenbergi	Not found
